# Integrated analyses reveal evolutionarily conserved and specific injury response genes in dorsal root ganglion

**DOI:** 10.1038/s41597-022-01783-8

**Published:** 2022-11-02

**Authors:** Lian Xu, Zhifeng Chen, Xiaodi Li, Hui Xu, Yu Zhang, Weiwei Yang, Jing Chen, Shuqiang Zhang, Lingchi Xu, Songlin Zhou, Guicai Li, Bin Yu, Xiaosong Gu, Jian Yang

**Affiliations:** 1 Key Laboratory of Neuroregeneration, Ministry of Education and Jiangsu Province, Co-innovation Center of Neuroregeneration, NMPA Key Laboratory for Research and Evaluation of Tissue Engineering Technology Products, Nantong University, 19# Qixiu Road, Nantong, Jiangsu 226001 China; 2 Nanjing University of Chinese Medicine, Nanjing, China; 3 Nantong Institute of Genetics and Reproductive Medicine, Affiliated Maternity and Child Healthcare Hospital of Nantong University, Nantong, Jiangsu China

**Keywords:** Regeneration and repair in the nervous system, Peripheral nervous system

## Abstract

Rodent dorsal root ganglion (DRG) is widely used for studying axonal injury. Extensive studies have explored genome-wide profiles on rodent DRGs under peripheral nerve insults. However, systematic integration and exploration of these data still be limited. Herein, we re-analyzed 21 RNA-seq datasets and presented a web-based resource (DRGProfile). We identified 53 evolutionarily conserved injury response genes, including well-known injury genes (*Atf3*, *Npy* and *Gal*) and less-studied transcriptional factors (*Arid5a*, *Csrnp1*, *Zfp367*). Notably, we identified species-preference injury response candidates (*e.g. Gpr151*, *Lipn*, *Anxa10* in mice; *Crisp3*, *Csrp3*, *Vip*, *Hamp* in rats). Temporal profile analysis reveals expression patterns of genes related to pre-regenerative and regenerating states. Finally, we found a large sex difference in response to sciatic nerve injury, and identified four male-specific markers (*Uty*, *Eif2s3y*, *Kdm5d*, *Ddx3y*) expressed in DRG. Our study provides a comprehensive integrated landscape for expression change in DRG upon injury which will greatly contribute to the neuroscience community.

## Introduction

**Fig. 1:**
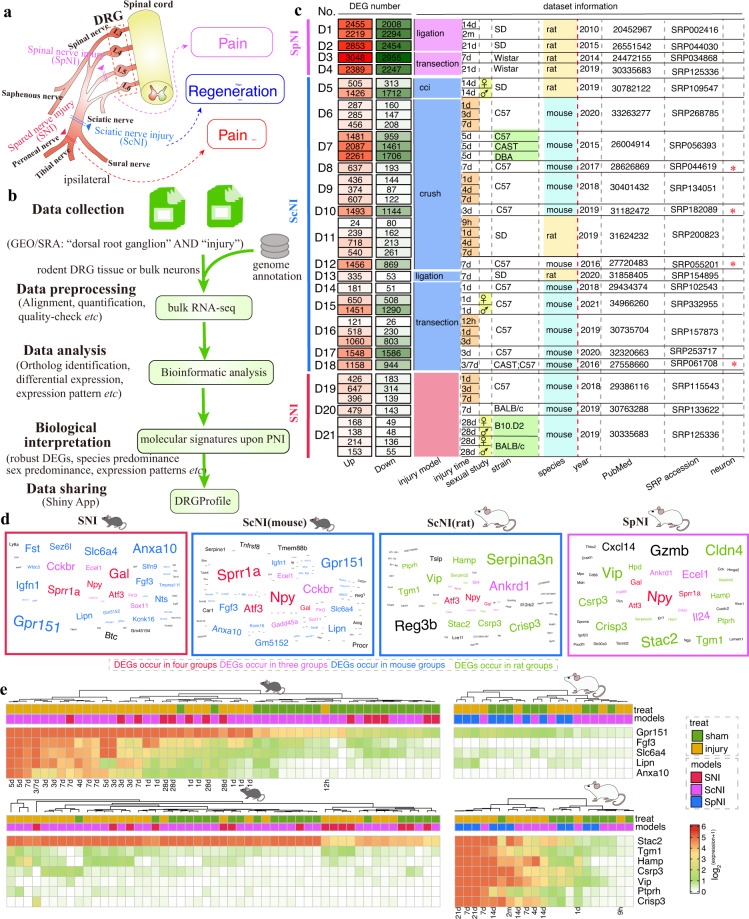
Analysis of the top 15 up-regulated genes in comparisons of injured rodent DRG with the control reveals species-preference in response to nerve injury. (**a**) The schematic illustration for peripheral nerve injury models employed in collected RNA-seq datasets. (**b**) The schematic illustration of the data processing in this study. (**c**) The number of differentially expressed genes (DEGs) and detailed information of 20 studies (21 datasets). Star indicated bulk RNA-seq of sensory neurons. cci, chronic constriction injury; d: days, h: hours, m: months; SD, Sprague-Dawley; CAST, CAST/Ei; C57: C57BL/6. (**d**) The wordcloud plots showed the frequency of the top 15 ranked up-regulated DEGs from each comparison within four groups (SNI and ScNI from mice and ScNI and SpNI from rats). Red-labeled genes indicated occurrence in four groups; pink-labeled genes indicated occurrence in three groups; blue-labeled genes indicated occurrence only in mouse groups; green-labeled genes indicated occurrence only in rat groups. (**e**) The expression of genes from mouse-preference (top panel) or rat-preference (bottom panel) in response to injury across each dataset. Only injured timepoints in the injury group from mice (top panel) or rats (bottom panel) were shown.

Unlike extremely limited regenerative capacity in CNS neurons upon injury, neurons in the peripheral nervous system (PNS) could regrow damaged axons and reinnervate targets^1^. However, this process is often incomplete and leads to sensory dysfunction and neuropathic pain (NP) in humans^2^. Understanding the cellular and molecular changes of damaged neurons responding to axonal injury is key to developing effective therapies against nerve injury and NP^1,2^. Dorsal root ganglion (DRG) neurons are unique in morphology with the cell body in the spinal nerve and axons bifurcate into a peripheral branch and a central branch, connecting peripheral target tissues and the spinal cord and conveying sensory information^3^. Besides, DRG neurons have a phenomenon called “conditioning” that activation of a transcriptional program after peripheral nerve injury (PNI) permits a robust regenerative response to the second insult of either the peripheral or central axon^4^. Current knowledge about molecular and cellular mechanisms on axon regeneration and NP development was most identified from rodent models. The common axonal injury models in rodents include damage (*e.g*. transection, ligation, crush) of two of three terminal branches of the sciatic nerve (SNI, leaving the sural nerve intact), spinal nerve (SpNI) and sciatic nerve (ScNI) (Fig. 1a). The former two are most used to establish NP models while the last specifically crush of the sciatic nerve is used to establish nerve regeneration models^5,6^.

Microarray and RNA-seq, together with the recent advance of the single-cell transcriptome (scRNA-seq) have provided genome-wide profiles of genes at tissue or cell resolution in the development and disease^7,8^. Extensive studies employed microarray and/or RNA-seq or scRNA-seq of DRGs have identified a variety of key molecules in regard to regeneration or NP in mice and/or rats under similar or different PNIs^4,9–30^, such as regeneration-associated genes (RAGs), *Atf3*, *Jun*, *Hsp27*, *Sprr1a*, *Gap43*. However, systematic integration and exploration of these public data are still limited. In addition, genome-wide comparison for identification of molecular similarities in DRG upon distinct axonal injuries (SNI, ScNI, SpNI) is also rare. A pilot study has integrated several microarrays of rat DRGs at different injured timepoints upon PNIs and performed a systems-level analysis of the regulatory network involved in axon regrowth after injury^31^. Species difference in response to nerve injury has been investigated between Schwann cells from human and mice^32^. Acting as the most commonly used pre-clinical injury models, genome-wide molecular evolutionarily conserved, and the difference between rats and mice in response to axonal injury is still being rarely discussed. A better understanding of molecular similarity and differences responding to nerve injury in these pre-clinical animals may contribute to screening more effective targets in the development of drugs for neural repair and NP.

To initially explore these, we performed a thoroughly integrated analysis of public expression profiles of rodent DRGs upon nerve injury in this study. Considering the limitations of microarray depending on prior probe design and inconsistency of array platforms and low accuracy in low expressed genes^8^, we only collected and re-analyzed public RNA-seq datasets of rodent DRGs upon PNIs (including SNI, ScNI, and SpNI) using a universal standard. Datasets with a low-quality were discarded and 21 public RNA-seq datasets from 20 independent studies^4,10–15,17–29^ were finally kept for the whole analysis. To aid in interpreting these data, we also re-analyzed other datasets (including profiles of sensory neurons upon injury^14^, profiles of sciatic nerve upon crush injury^33^, and long-term time-series profiles of the spinal cord in development^34^ and injury^35^). We defined a robust differential expression gene (DEG) which showed differential expression in most injured groups than the corresponding control group (sham-operated or naïve groups). We detected 53 robustly up-regulated genes across species and injury models, including well-documented RAGs (*Atf3*, *Npy*, *Gal*) and other less-studied transcriptional factors (TF, e.g. *Csrnp1*, *Arid5a*, *Zfp367*). We also found that some well-documented genes with dramatic changes upon injury presented species-preference manners at the transcriptional level, such as *Hamp*, *Vip*, *Serpina3n* in rats, and *Gpr151* in mice. In addition, we also explored gene expression patterns specifically pre-regeneration and regenerating related genes by re-analysis of the time-series RNAseq datasets. We also initially explored sex differences in response to injury and identified four male-specific markers expressed in DRG. Finally, we present a web-based application for exploring gene expression change in rodent DRGs upon nerve injury to aid the development of novel therapeutics for neural repair and neuropathic pain.

## Results

### Overview of public RNA-seq of rodent DRG tissue or sensory neurons under distinct PNIs

We collected 21 bulk RNA-seq datasets of rodent DRGs (tissue or sensory neurons) upon PNI (SpNI, ScNI, and SNI) from 20 studies with injury-time points ranging from hours (3 h, 9 h, 12 h) to months (2 m) which focused on nerve regeneration or neuropathic pain^4,10–15,17–29^ (Fig. 1 and Supplementary Table 1). Only libraries related to injury or control were kept for each dataset (Supplementary Table 2 available at Figshare). We next performed a uniform pipeline to re-analyze, including quality-control, mapping against the reference genome, gene expression quantification, and differential expression (DE) analysis (Fig. 1b and Method). The ScNI is the most investigated rodent model with thirteen datasets and species including rats and mice. Three datasets and four datasets investigated profiles of DRG in SNI (mice) and SpNI (rats) models, respectively. To explore molecular characters and expression patterns in DRG upon axonal injury, we only investigated time points with significant up-regulation of *Atf3*, a neuronal injury marker^36^. DE analysis was performed using the injury group compared to the sham or naïve group (defined as the control group) and results showed that *Atf3* was significantly up-regulated at as early as 9 h, thus we focused on injured timepoints ranging from 9 h to 2 m. 1d, 3d,and 7d (days) post-injury are the most investigated time points. Five datasets investigated time-series (at least three injured time points were considered) within the first seven days upon injury, species strains, and gender differences were investigated in two datasets and four datasets respectively. DE analysis showed a vast of molecular changes in the SpNI model and a small number of DEGs in the SNI model, while varied DEG numbers were detected in the ScNI compared to the corresponding control groups (Fig. 1c). We next ranked DEGs in each comparison of datasets by adopting the π-value metric^37^ which considers both significance (*e.g*. adjusted *P*-value) and log-transformed fold-change (LFC) for datasets with biological replicates while only LFC for datasets without biological replicates (Supplementary Figs. 1 and 2). We found three well-known injury-induced genes (*Atf3*, *Npy*, *Gal*) that showed a high frequency of the top-ranking DEGs (Fig. 1d). However, we found several genes with a high frequency of the top-ranking DEGs that showed species-preference manners featured with a dramatic change in mice (rats) while a small change in rats (mice) upon injury, such as *Gpr151*, *Anxa10*, *Slc6a4*, *Lipn*, *Fgf3* in mice, while *Stac2*, *Ptprh*, *Hamp*, *Crisp3*, *Tgm1*, *Vip*, *Csrp3* in rats (Fig. 1d,e). We found a similar low expression level of these injury-induced genes in the control group from mice and rats, except for *Stac2*, while a different response specifically in the late post-injury (*e.g*. 3d~7d) with either up-regulated in mice or rats. Some of which have been documented with roles (lipid metabolism: *Lipn*; calcium channel related: neuronal *Stac2*; immune and inflammation: *Anxa10*, *Ptprh* (also known as SAP-1)^38^, *Crisp3* ^39^; actin cytoskeleton: *Csrp3*; neuroprotective peptides: *Vip* and *Hamp*; cell proliferation: *Tgm1*) in axonal injury, axonal regeneration (*Gpr151*, *Slc6a4*), or neuropathic pain (*Gpr151*^40^, *Anxa10*^41^). We also found that mouse-preference top ranking genes (*Anxa10*, *Slc6a4*, and *Lipn*) showed dramatic changes in mouse sensory neurons collected from in *vivo* post-injury than that from in *vitro* after plating^4^ (Supplementary Fig. 3).

### Identification of robust differential expression genes (DEGs) in response to axonal injury

To systematically investigate expression patterns of DEGs in either SNI, ScNI, or SpNI from rats and mice compared to the control group, we adopted a uniform threshold (adjusted *P*-value ≤ 0.05 and |log_2_^fold-change^| ≥ log_2_^1.5^) and calculated the frequency of DEGs. DEGs with a frequency ≥50% of compared groups in either up-regulated or down-regulated were defined as robustly up or down-regulated. We next deeply explored robust DEGs in DRG from mice and rats upon SNI, ScNI, and SpNI, separately.

**Fig. 2:**
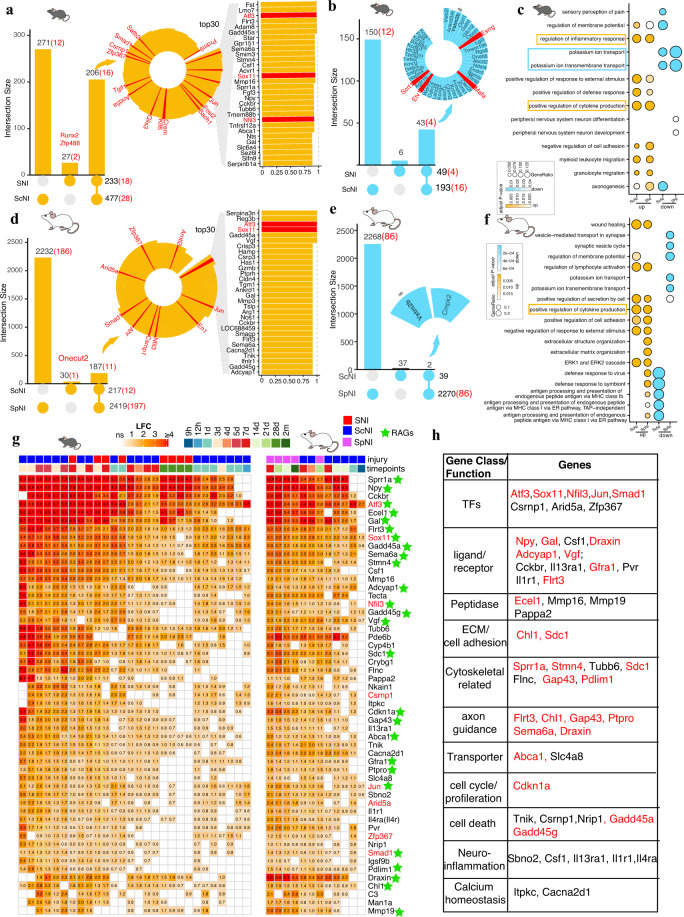
Robust and conserved DEGs in rodent DRGs upon PNI. (**a**) Upset plots showed robust up-regulated DEGs upon SNI and ScNI mouse models. The Circular barplots showed the overlapped DEGs and 30 top-ranking genes were shown in the regular barplots. Red text indicated transcription factors. (**b**) Robust down-regulated DEGs upon SNI and ScNI mouse models. (**c**) Compared GO(BP) enrichment in either robust up-regulated or down-regulated in ScNI and SNI mice models. Top five categories were shown. (**d**) Robust up-regulated DEGs upon ScNI and SpNI rat models. (**e**) Robust down-regulated DEGs upon ScNI and SpNI rat models. (**f**) Compared GO (BP) enrichment in either robust up-regulated or down-regulated in ScNI and SpNI rat models. Top five categories were shown. (**g**) Fold-change of 53 robust up-regulated DEGs commonly in distinct PNI models. Green star indicated documented RAGs. Numbers in the cells were log-transformed fold-change (LFC) compared to the corresponding control groups. LFC of genes without significance was not shown. (**h**) Functional annotation of commonly up-regulated genes in distinct PNI models by manual reviews and functional annotation.

Eight comparisons from three studies investigated profiles of mouse DRG upon SNI regarding a time-series (1d, 3d,and 7d), strain, and gender (female and male). We identified 233 genes that were robust up-regulation and 49 genes that were robust down-regulation, respectively (Fig. 2a,b, and Supplementary Table 3 available at Figshare). Of which, 18 and 4 transcription factors (TFs) showed robust up-regulation and down-regulation respectively. Twenty comparisons from eleven studies investigated expression profiles of mouse ScNI, including the conditions of time-series, strain, and sex at tissue or neuron level. We identified 477 robustly upregulated genes and 193 robustly downregulated genes respectively (Fig. 2a,b and Supplementary Table 3 available at Figshare). Of which, 28 and 16 TFs were robustly up-regulated and down-regulated respectively. Analysis of robust DEGs in mice upon SNI and ScNI showed 206 (88.4% of mouse SNI) and 43 (87.8% of mouse SNI) DEGs were commonly upregulated and downregulated respectively (Fig. 2a,b and Supplementary Table 4 available at Figshare). Functional enrichment analysis showed robustly up-regulated genes in mice upon injury were significantly enriched in terms related with the inflammatory response (*e.g*. cytokine production, cell adhesion, migration) while down-regulated genes were consistently involved in potassium ion transport (Fig. 2c).

Seven comparisons from three studies investigated profiles of rat DRG upon ScNI. We identified 217 robust upregulated and 39 robust downregulated DEGs respectively (Fig. 2d,e and Supplementary Table 3 available at Figshare). Of which, 12 TFs showed robust up-regulation. Five comparisons from four studies investigated profiles of rat DRG upon SpNI. Different with the number of robust DEGs detected in SNI and ScNI models, a larger number of DEGs (2,419 up-regulation and 2,270 down-regulation) were identified in the SpNI model (Fig. 2d,e and Supplementary Table 3 available at Figshare). A relatively large number of up-regulated DEGs (187, 86.2% of rat ScNI) were detected in both rat ScNI and SpNI models while a small number of down-regulated DEGs (2, 5.1% of rat ScNI, Fig. 2d,e and Supplementary Table 4 available at Figshare). Functional enrichment analysis showed the most enriched terms in robust up-regulated genes, including wound healing, immune cell activation, cell adhesion, and cytokine production (Fig. 2f).

To further screen rodent conserved injury-related genes, robust DEGs occurred in both rat and mouse SNI, ScNI, and SpNI models were selected. We observed a certain number of up-regulated genes (53 DEGs) while only *Vstm2b* (V-set and transmembrane domain-containing protein 2B) showed commonly down-regulated (Fig. 2g). We manually reviewed literature related to these genes to infer potential functions in nerve injury by searching against the PubMed. Twenty-six (49%) out of fifty-three robustly upregulated DEGs were documented as the RAGs, including TFs (*Atf3*, *Sox11*, *Jun*, *Nfil3*, and *Smad1*), cytoskeletal related proteins (*Sprr1a*, *Stmn4*, *Sdc1*, *Gap43*, and *Pdlim1*), ligand/receptors (peptides/neurotrophic factors: *Npy*, *Gal*, *Adcyap1*, and *Vgf*; GDNF receptor: *Gfra1*), peptidase (*Ecel1* and *Mmp19*), ECM and cell adhesion (*Chl1* and *Sdc1*) (Fig. 2h). Long-term spinal cord development profiles (covering embryonic, neonatal, young, and adult stages)^34^ and embryonic DRG profiles^4^ showed some of these genes were development-related genes, such as *Sox11*, *Draxin*, *Flrt3*, *Sdc1* (Supplementary Figs. 4 and 5). In addition, we found that 39 out of 53 genes showed significant up-regulation in injured DRG neurons featured with a high expression of *Atf3*^14^, suggesting most of these genes may be related to injured neurons response, specifically two less-studied TFs (*Arid5a* and *Zfp367*) (Supplementary Fig. 6). We next investigated the expression of these robust DEGs in central nerve injury based on previously long-term RNA-seq of spinal cord hemi-transection injury^35^. The results showed most genes presented immediate injury response (*e.g*. 3 h), including TFs (*Arid5a*, *Csrnp1*), suggesting their potential role as an early injury marker. *Arid5a* has dual roles acting as a TF and RNA-binding protein, involved in the development and immune regulation respectively^42^. *Csrnp1* (also known as AXUD1), a TF, could be up-regulated in inflammatory insult to regulate matrix metalloproteinases in human chondrocytes^43^. In addition, *Csrnp1* has also been proven with roles in development, including cephalic neural progenitor proliferation and survival in zebrafish and neural crest formation^44,45^. Different from *Arid5a* and *Csrnp1* in spinal cord injury, we found *Zfp367* (zinc finger protein 367) was only up-regulated at 3d post-injury (Supplementary Fig. 7). Consistently upregulated of these three uncharacterized TFs, particular two immediate response TFs (*Arid5a* and *Csrnp1*), in peripheral nerve injury and central nerve injury, highlighted their importance in axonal injury and required further investigation.

### The distinct molecular response between rat and mouse upon ScNI

**Fig. 3:**
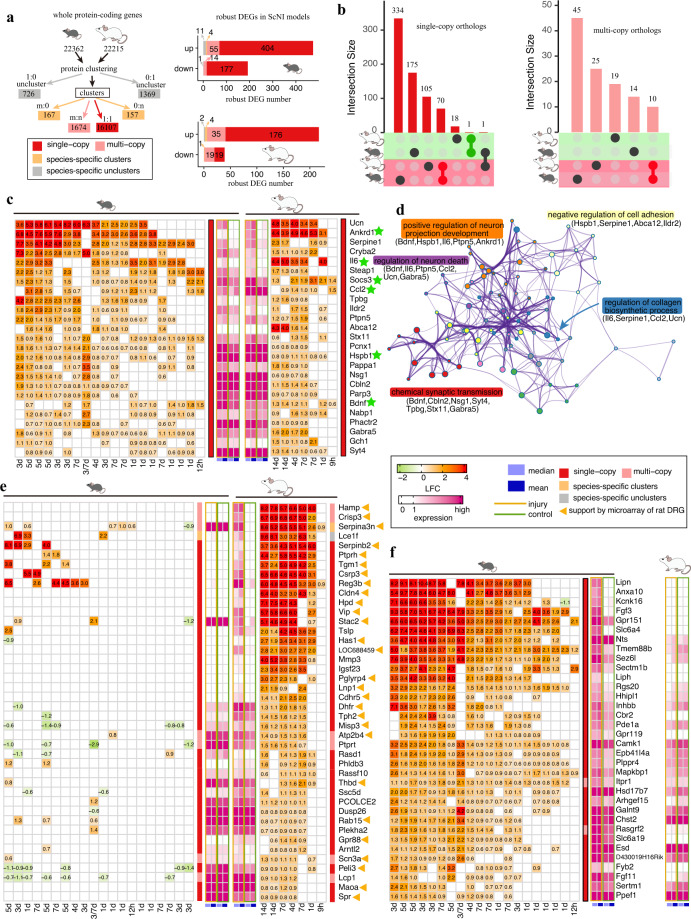
Evolutionary conserved and specific robust DEGs in rats and mice upon ScNI. (**a**) Summary of the protein clustering analysis between rat and mouse (left) and the number of DEGs in each category in rats and mice upon ScNI (right). (**b**) UpSet plots showed the relationship of robust DEGs belonging single-copy (left) or multiple-copy (right) between rats and mice. Fold-change and expression of additional 25 common robust DEGs (**c**) In rats and mice upon injury and GO enrichment (**d**). (**e**) Fold-change and expression of 42 rat-preference candidates. (**f**) Fold-change and expression of 34 mouse-preference candidates. The expression level of genes in the injured DRG and control was indicated by the average and median of normalized expression values.

Next, we focused on distinct molecular responses to the nerve injury between rats and mice which were the two most widely used pre-clinical animal models in studying neural repair and NP. Considering datasets of mice and rats were both available in ScNI models (Fig. 1b), we systematically investigated robust DEGs in ScNI models at species-level. To avoid bias caused by gene nomenclature between mouse and rat or some inconsistent gene names from different databases (such as NCBI and Ensembl), we first performed protein clustering analysis to detect orthologous relationships between rat and mouse, and identified 16,107 single-copy orthologs and 1,674 multi-copy orthologs, and 324 divergent or species-specific clusters in either rat (157 clusters) or mouse (167 clusters) (Fig. 3a). We found most robust DEGs were the single-copy orthologs, followed by the multi-copy orthologs between mouse and rat (Fig. 3a). We found 704 single-copy orthologs showed robust DEGs with 581 (up-regulation: 404 genes, down-regulation: 177 genes) in mice and 195 (up-regulation: 176 genes, down-regulation: 19 genes) in rats (Fig. 3a and Supplementary Table 4 available at Figshare).

We found that 70 genes from single-copy orthologs and 10 genes from multi-copy orthologs were detected as robust up-regulated DEGs in rat and mouse ScNI models (Fig. 3b). Forty-five out of seventy and eight out of ten genes have been detected above as robust DEGs in PNI (Fig. 2g). In addition, we found twenty-five genes (including RAGs: *Socs3*, *Hspb1* (also known as *Hsp27*), *Ccl2*, *Ankrd1*, *Il6* and *Bdnf*) and two genes (*Tubb2b* and *Serpinb1a*) from single-copy and multi-copy orthologs detected as robustly up-regulated DEGs in ScNI respectively (Fig. 3c). These DEGs were significantly enriched in terms related with synaptic transmission (*Bdnf*, *Gabra5*, *Nsg1*, *Syt4*, *Tpbg*, *Stx11*, and *Cbln2*), regulation of collagen biosynthetic process (*Il6*, *Serpine1*, *Ccl2*, and *Ucn*), regulation of neuron death (*Bdnf*, *Il6*, *Ptpn5*, *Ccl2*, *Ucn*, and *Gabra5*), and regulation of neuron projection development (*Bdnf*, *Hspb1*, *Il6*, *Ptpn5*, *Ankrd1*) (Fig. 3d). Corticotropin-releasing factor (CRF) family contains four members (*Crh*, *Ucn*, *Ucn2*, and *Ucn3*) in mammals and binds to CRF receptors (*Crhr1* and/or *Crhr2*) involved in stress response^46^. It has been shown that *Ucn2* is rapidly expressed at the neuromuscular junction (NMJ) after α-Latrotoxin induced NMJ degeneration and Ucn2-Crhr2 axis may be a novel role in NMJ regeneration^47^. We found a low expression of *Ucn2* in the normal condition (sham), while dramatically increased in sciatic nerve (SN)^33^ at 1d post-ScNI (Supplementary Fig. 8). However, a relative low-expression and small change of *Ucn2* in DRG tissue or neurons post-ScNI. Interestingly, different from the expression pattern of *Ucn2*, we found a low expression of *Ucn* in SN post-ScNI and showed robust up-regulation in DRG tissue and neuron (>80 fold-change) post-ScNI. Furthermore, the expression profiles of sensory neurons collected from plating in *vitro* and in *vivo* post-ScNI showed a larger change of *Ucn* expression in *vivo* than that from in *vitro*, while a larger change of *Ucn2* expression in *vitro* than that from in *vivo* (Supplementary Fig. 8). These results suggested injury-induced *Ucn* and *Ucn2* have undergone subfunction with distinct expression patterns and tissue-specific in response to ScNI. Except for other studied genes in nerve injury (*Parp3*^48^, *Gch1*^49^), we also noticed two genes (nucleic acid binding protein 1 (*Nabp1*) and six transmembrane epithelial antigen of the prostate 1 (*Steap1*)) featured with a robust up-regulation in DRG and a dramatic increase (*Nabp1*: 7.7-fold; *Steap1*: 114.5-fold in SN at 1d post-ScNI) in SN upon ScNI (Supplementary Fig. 9) while studies related their expression and function in axon injury and repair still be scarce.

Due to a small number of consistent down-regulated robust DEGs, we mainly focused on “species-preference” (here defined as the difference in gene expression change upon injury between rats and mice) up-regulated robust DEGs upon ScNI in rats and mice. We initially detected 334 genes from single-copy and 45 genes from multi-copy orthologs that showed mouse-preference robust up-regulated genes, and 106 genes (including 1 gene up-regulated in rats while down-regulated in mice) from single-copy and 25 genes from multi-copy orthologs that showed rat-preference robust up-regulated genes (Fig. 3b and Supplementary Table 5 available at Figshare). We found some immune and inflammation-related genes (*e.g. Trem2*, *Fcgr2b*) present in the “mouse-preference” group, which may be caused by the limited datasets (only three datasets including one without biological replicate), the weak threshold (occurrence at least 50% of the compared groups), and un-balanced timepoints in rats, specifically 3d~7d post-injury when the proliferation of the immune cells (*e.g*. macrophage) in DRG^11^. Therefore, we only focused on those genes that featured a low expression or small changes in either mice or rats and integrated our previous microarray data of rat DRGs (1d, 4d, 7d,and 14d post-ScNI)^30^ and bulk RNA-seq of rat spinal cord injury dataset^35^ to avoid misinterpretation. Finally, we manually screened 42 candidate rat-preference response DEGs and 28 of which could be well supported by the array data^30^ (Fig. 3e). We noticed rat-preference DEGs with dramatic changes upon injury included genes that have been reported and validated in rat PNI models, including *Hamp*^50^, *Serpina3n*^51^, *Tgm1*^52^, *Csrp3*^53^, *Reg3b*^54^, *Vip*^52^, *Tslp*^55^ and *Atp2b4* (PMCA4)^56^. Thirty-four mouse-preference response DEGs were further screened, including documented genes in mouse nerve injury models, *Slc6a4*/SERT1^57^, *Inhbb*^27^, *Fgf3*^58,59^, *Plppr4*/PRG-1^60^, *Gpr151*, and *Anxa10* (Fig. 3f).

**Fig. 4:**
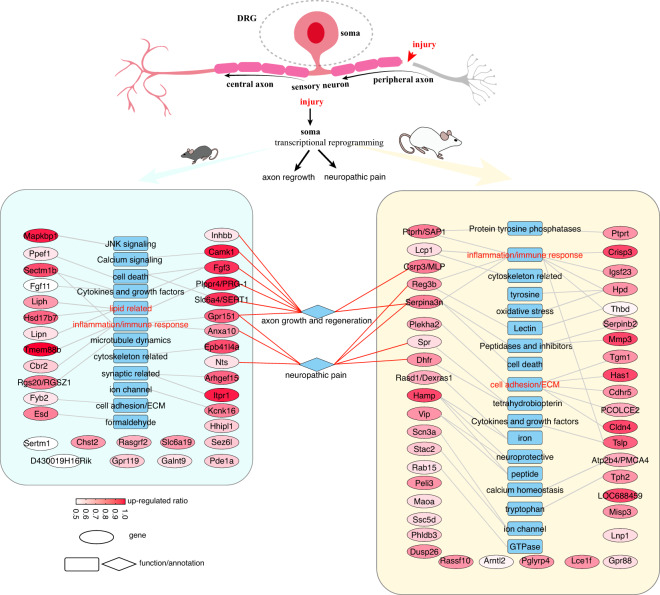
Summary of potential functions and annotations of mouse-preference and rat-preference candidates upon PNI. Blue rectangles and diamonds indicated the potential function or annotation of genes. Ellipse indicated genes and color indicated up-regulated ratio.

We found potential roles of most genes have not been well explored in nerve injury although their dramatic and robust changes upon injury, such as *Crisp3*, *Stac2*, *LOC688459* in rats and *Tmem88b* in mice (Fig. 4). Of rat-preference response genes, *Serpina3n* diverged in sequence between rat and mouse, showed a low expression in rats but a high expression in mice under the normal condition, and presented a distinct response upon injury in mice and rats (significant up-regulation in rats but small difference in mice, Fig. 3e). This gene has been validated up-regulated at mRNA level in rats and mice upon nerve injury but rats showed a larger difference and exogenous delivery in mice could attenuate neuropathic pain by inhibiting T cell-derived leukocyte elastase^51^. Different with the expression pattern of *Serpina3n*, we found two other peptidase/inhibitors (*Serpinb2* and *Mmp3*) showed low expression in rats and mice under the normal condition but robustly induced only in rats upon injury (Figs. 3e and 4). We also found five genes (*Tgm1*, *Has1*, *Cdhr5*, PCOLCE2, and *Cldn4*) involved in cell adhesion or ECM organization. *Cldn4* belonging to the tight junction protein, has been shown to protect against acute lung injury^61^. We found some genes related with metabolism that showed rat-preference, including iron homeostasis (*Hamp*, *Rasd1*/*Dexras1*), tetrahydrobiopterin production (*Spr*, *Dhfr*)^49^, tyrosine (*Hpd*^62^), tryptophan (*Tph2*), and calcium homeostasis (*Atp2b4*/PMCA4^56^). *Hamp*, induced by inflammation stimulus, has been validated up-regulation in rat DRG upon ScNI and could be transported into regenerating axons upon ScNI and effect neuroprotective by reducing iron in rat primary cortical neuron against hemin and iron-mediated neurotoxicity^50,63^. In addition, we also found genes related with oxidative stress (*Hpd*), inflammation and immune response (*Ptprh*/SAP1, *Lcp1*, *Reg3b*, *Serpina3n*, *Crisp3*, *Igsf23*, *Hpd*, *Thbd*, *Tslp*) showed a rat-preference response (Fig. 4). *Csrp3*, previously regarded as a muscle-specific protein (known as MLP), has been proven as induced in rat retinal ganglion cells while neither in mouse retina nor in DRG neurons upon axotomy, indicating it’s a rat-specific injury response gene^64^. Different expression changes of MLP in mice and rats upon nerve injury allow an alternative experiment to confirm its role in promoting nerve regeneration by ectopic MLP expression in mice^64^. And further functional experiments confirmed the pro-regeneration role of *Csrp3* by acting as a cross-linker, facilitating filopodia formation and increasing growth cone motility^64^.

Of mouse-preference candidates, we found genes related with inflammation and immune response (*Gpr151*, *Anxa10*, *Cbr2*, *Tmem88b* and *Sectm1b*), lipid (*Plppr4*/PRG-1, *Hsd17b7*, *Liph* and *Lipn*), cell death (*Ppef1*, *Fgf3*), calcium signaling (*Camk1*), cytoskeleton related (*Epb41l4a*), microtubule dynamics (*Rgs20*/RGSZ1), synaptic related (*Arhgef15*, *Itpr1*), and neuropeptide (*Nts*) were present (Fig. 4). Some genes showed specifically induced in mice upon injury, such as *Lipn*, *Anxa10*, *Kcnk16*, *Fgf3*, *Slc6a4*. Accumulated triglyceride storage lipids in neurons upon axotomy impede CNS regeneration^65^. *Lipn*, an acid lipase converting triacylglycerol into fatty acid, has been reported expressed in skin and involved in epidermal differentiation and low expression in other tissue^66^. Indeed, we found low expression of *Lipn* in DRG and spinal cord development. It could be robustly induced in injured neurons in mice at 3d~7d post-ScNI (Fig. 3f). *Plppr4*, a new neuronal phospholipid phosphatase, could attenuate phospholipid-induced axon collapse, promoting axon growth and regenerative sprouting in the hippocampus^60^. Analysis expression of these lipid-related genes (*Plppr4*, *Lipn*, *Liph*, and *Hsd17b7*) in rat DRGs or spinal cord upon PNS or CNS injury showed extremely low-expression or down-regulated expression patterns, indicating different responses upon injury in lipid metabolism between mouse and rat.

### Temporal expression signatures of DEGs upon PNI

**Fig. 5:**
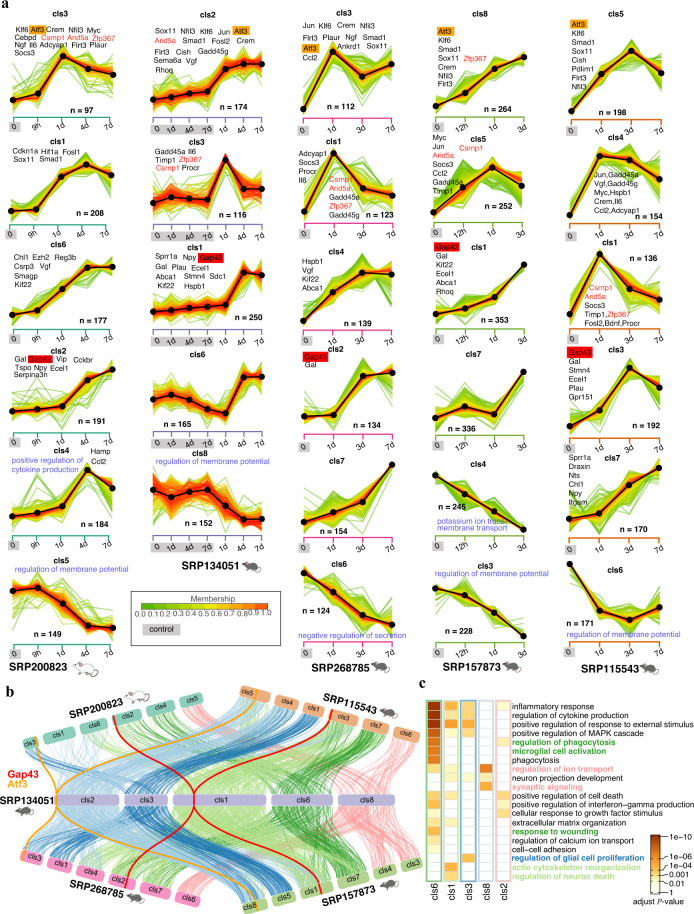
Clustering analysis of five time-series datasets reveals expression patterns of genes related with pre-regeneration and regeneration. (**a**) Major gene clusters in five time-series datasets. Major clusters with genes documented related with axon regeneration or with small fluctuations in the contralateral across time points were shown. Expression patterns of all clusters in each dataset were shown in Supplementary Fig. 10. Each line indicated a gene and color indicated membership values. (**b**) Gene relationship of other four time-series datasets relative to the reference dataset. Gene was ordered in each cluster by membership values. Each line indicated a shared gene between a cluster from the reference dataset and a cluster from the other datasets. Only number of shared genes greater than 10 was shown. Line color indicates distinct clusters from the reference dataset. (**c**) GO enrichment of genes from five clusters in the reference dataset. Color indicated adjust *P*-value of enrichment analysis.

Four datasets and one dataset explored time-series of expression profiles of DRG upon ScNI (including timepoints, 9 h, 12 h, 1d, 3d, 4d,and/or 7d) and SNI (1d, 3d,and 7d) respectively. To understand gene expression patterns of DEGs upon injury, we employed the Mfuzz package to cluster DEGs into eight groups for each dataset (major clusters were shown in Fig. 5a and all clusters were shown in Supplementary Fig. 10; Supplementary Table 6 available at Figshare). *Atf3* and *Gap43* are well-known two markers for neurons at injured and regenerating states respectively. We found *Atf3* and *Gap43* present in the groups with expression patterns significantly up-regulated at as early as 1d and 3d respectively (Fig. 5a). Of the *Atf3* contained clusters, we found other five genes present in these five datasets, including RAGs (*Klf6*^67^, *Nfil3*, *Flrt3*) and other genes (*Vash2*, *Srxnh1*). *Vash2* (vasohibin-2), a component of tyrosine carboxypeptidase, regulates neuron differentiation by affecting detyrosinated α-tubulin levels^68^. We also found that its expression decreased along the spinal cord and DRG development. *Srxn1* (Sulfiredoxin1), an endogenous antioxidant protein, could prevent cell oxidative stress and has been reported as up-regulation in cerebral ischemia/reperfusion (I/R) injury with neuroprotective effects^69^. Analysis of other well-known pro-regenerative TFs (*e.g. Crem*, *Myc*, *Smad1*, *Sox11*, *Jun*, Fig. 5a) indicated that expression of these genes also presented initiation up-regulation at 1d upon injury (called “pre-regenerative” phase as we described before^30^), suggesting 1d is a key timepoint for regenerative-related transcriptional reprogramming. We also found three TFs (*Arid5a*, *Zfp367*, and *Csrnp1*) discussed above showed co-occurrence and presented a similar expression pattern with those pro-regenerative TFs (Fig. 5a), supporting their importance in axonal injury. We also checked other known RAGs (*e.g. Il6*, *Hspb1*, *Adcyap1*, *Cckbr*, *Npy*, *Ecel1*) in each cluster from each dataset and showed most RAGs were up-regulated at 1d and/or 3d~7d (Fig. 5a). Next, we explored the relationship of clusters among five datasets. Considering the high membership of genes belonging to the cluster from SRP134051 and including the corresponding control group in each injured timepoint, we selected it as a reference and calculated the overlapped genes from each cluster between this reference and other datasets (only connected gene number ≥10 was shown among major clusters, Fig. 5b and Supplementary Table 7 available at Figshare). The result showed a high consistency of clusters between the reference and other datasets, specifically in mice (Fig. 5b). To explore the potential functions of genes in these clusters, we conducted a functional enrichment analysis of genes in the clusters from the reference dataset. The results showed genes with expression decreased upon injury (cluster(cls) 8 of the reference dataset) were involved in the regulation of ion transport and synaptic signaling (Fig. 5c). Genes (e.g. *Il6*, *Vgf* and other pro-regenerative TFs) with high expression at 1d (cls2 and cls3) showed most enriched in terms regulation of MAPK cascade, regulation of response to external stimulus, and regulation of glia cell proliferation (Fig. 5c). Genes (e.g. *Sprr1a*, *Gpr151*, *Nts*, *Gal*, *Stmn4*, *Plau*, *Abca1*, *Npy*) in the cluster contained *Gap43* (cls1) were most enriched in actin cytoskeleton reorganization while genes (*e.g. Aif1*(IBA1), *Cd68*, *Trem2*, *P2ry6*) with another similar trend (cls6) were mostly involved in immune and inflammatory response (Fig. 5c), suggesting 3d/4d was a key timepoint for regeneration and inflammation and also the importance of neuron-immune contribution to axonal regeneration.

### Sexual dimorphism of DEGs upon nerve injury

**Fig. 6:**
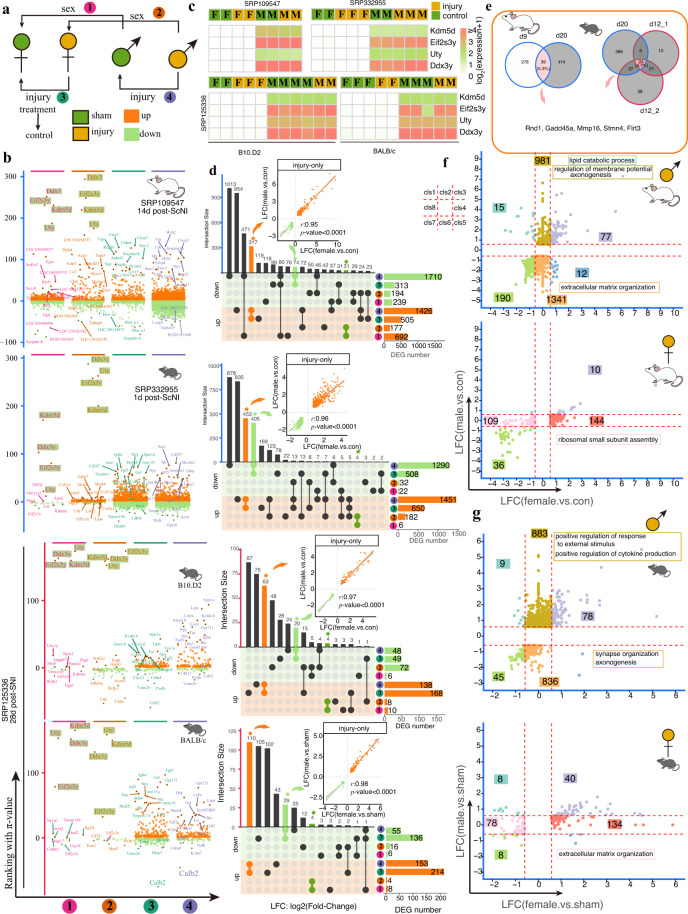
A relatively large number of DEGs in males responding to sciatic nerve injury and identification of four male-specific markers expressed in DRG. (**a**) Schema of compared groups. (**b**) Manhattan plots showed DEGs in four compared groups. Orange points indicated significantly up-regulated genes while green indicated significantly down-regulated genes. Gene ranking was ordered based on π-value. Top 15 up-regulated and top 5 down-regulated genes were labeled. Four male-specific markers were shaded. (**c**) Expression of four male-specific markers in four datasets. (**d**) Upset plots of the relationship of DEGs identified from four compared groups in each dataset. (**e**) Venndiagram of common injury genes in rats and mice. (**f**,**g**) Scatter plots of male- or female-preference DEGs in rat and mice upon ScNI. Eight clusters with distinct colors were divided based on the cutoff of fold-change (1.5, red dotted lines). Number shaded with colorful boxes indicated DEG numbers in each cluster. Importantly enriched GO terms in a cluster were shown.

Sexual dimorphism of transcriptional changes in mouse or rat DRG at early or late upon nerve injury has been reported in several independent studies^15,16,23,26^. We re-analyzed four publicly available RNA-seq datasets from three studies^15,23,26^, including rats (14d post-ScNI) and mice (1d post-ScNI, and 28d post-SNI for strains (B10.D2 and BALB/c)). We performed the analysis by comparison between females and/or males with control and/or injury (Fig. 6a). We found a larger number of DEGs in the ScNI model (>800) than that in the SNI model (<400), and a larger number (>2-fold) of male DEGs than female DEGs in the ScNI model while a small difference (<2-fold) of that in the SNI model (28d) (Fig. 6b). Another study investigated sex difference in response to SNI in rat and showed large DEGs in injury group while also a small difference in number of DEG in female and male injury group compared to the control^16^. We found four male-specific genes (located in chromosome Y; *Uty*, *Eif2s3y*, *Kdm5d*, and *Ddx3y* (*Ddx3* for rat) were commonly present in the top 10 male up-regulated DEGs (ranking by π-value) compared with female in control or injury groups (Fig. 6b). Then, we used these four genes to infer sex in other datasets discussed in this study. Except for four studies (including one bulk sequencing of neurons^18^ (SRP182089) that was not separately discussed in this section), we showed that most studies employed males or at least mixed females and males in a study, but also existed three studies with inconsistent sex composition in other biological replicates or other treatment in a study (Supplementary Fig. 11). This may cause detected DEGs not only affected by treatment (*e.g*. injury in a study) but also sex itself differences. For example, the injury group from the study^21^ (SRP253717) was males but the control group was females and the most ranking down-regulated DEGs were male-specific genes (*Uty*, *Eif2s3y*, *Kdm5d*, and *Ddx3y*, Supplementary Fig. 2). Notably, we found larger sex-related DEGs in rats (692 genes) than in mice (≤10 genes) implicated by the small number of DEGs by comparing females and males in the control group (Fig. 6b,d). We divided these DEGs into three groups with a focus on DEGs related “injury-only” (Fig. 6d). Analysis of “injury-only” genes featured differential expression (consistent up/down-regulation) in females and males with injury treatment compared to the corresponding control group without any difference regarding sex. We found a strong significant correlation between these “injury-only” genes between females and males (*r* > 0.95 and *p*-value < 0.0001, Fig. 6d and Supplementary Table 8 available at Figshare), such as *Gadd45a*, *Mmp16*, *Stmn4*, *Flrt3* (Fig. 6e).

Next, we focused on the difference in either females or males in responses to injury. Considering the late injured timepoints (28d) and the small number of DEGs upon SNI injury, we only focused on two ScNI datasets. We divided DEGs of either female or male-preference based on their LFC and split into eight groups based on a 1.5-fold change and performed enrichment analysis (Fig. 6f and Supplementary Table 9 available at Figshare). The result showed genes up-regulated in males but not females in rats were mostly enriched in terms related with regulation of membrane potential axonogenesis while down-regulated in males but not females were mostly enriched in the extracellular matrix organization (Fig. 6f). Unlike in rats, genes with up-regulation in mouse males but not females were mostly enriched in terms related with positive regulation of response to external stimulus and positive regulation of cytokine production while down-regulated in male-only showed synapse organization axonogenesis were mostly enriched (Fig. 6g). The difference between rats and mice in sexual difference upon injury may be also related with their injured timepoints that the former was 14d post-ScNI (injury repair) while the latter was 1d post-ScNI (injury response).

## Discussion

Understanding the mechanism of axon regeneration, including the intrinsic growth ability and permissive microenvironment (*e.g*. extracellular matrix) of injured neurons upon PNS has provided insights into possible strategies for the treatment of CNS injury^1,70^. Transcriptomic studies of rodent DRGs (mainly mouse and rat) upon PNI have provided valuable insights into the transcriptional programs: downregulating genes related with neuronal activity (*e.g*. ion channel) along with neuronal maintenance genes; while upregulating pro-growth transcriptional factors and growth-associated proteins^1^. Though bulk transcriptome or single-cell transcriptome of post-mortem human DRGs have been studied as well as compared with mice^71,72^, human DRGs upon PNI still lack. As the most used pre-clinical animal models, although extensive studies have explored genome-wide expression profiles in either rats or mice DRG at tissue and/or single-cell levels upon PNI and several studies have indicated distinct expression signatures of individual genes between rats and mice in response to PNI^51,64^, systematic integration and comparison of response changes at the genome-wide molecular level still be limited. Understanding molecular similarity and difference in DRGs upon PNI across species may provide important new targets for translational approaches to treat nerve injury and NP. To achieve this, we firstly collected and performed a universal and systematic analysis by integrating public bulk RNA-seq on rat and/or mouse DRGs upon PNI and interpreted by integrating other datasets (*e.g*. spinal cord development^34^ and injury^35^, sciatic nerve upon crush injury^33^, high-coverage single-cell RNA-seq of sensory neurons^14^) and established a web application for convenient access data (DRGProfile).

We found a large number of DEGs upon SpNI while a relatively smaller number of DEGs in ScNI and SNI models. Further, we found a relatively larger number of DEGs in neurons than tissue upon the ScNI, except for a study that contained inconsistently sex composition^21^ (female in injury groups while male in control groups) and two sexual studies^15,26^, suggesting sex difference in response to ScNI is important and should be attended in designing the experiment. Analysis of occurrence frequency of top-ranking genes showed that some well-known genes (*e.g. Gpr151*, *Fgf3*, *Serpina3n*, *Vip*, *Hamp*) present species-specific manner. Cross-validation of multiple datasets across species, injury models, and similar or distinct injured timepoints, allowed us to screen robust injury response genes. We found a small number of down-regulated genes (*Vstm2d*) and identified 53 robust up-regulated genes. Most of them have been functionally explored, specifically those injury-induced genes that featured a dramatic change upon injury (*e.g. Atf3*, *Sprr1a*, *Npy*, *Cckbr*, *Ecel1*, *Gal*, *Flrt3*). In addition, we also noticed three less-studied TFs (*Arid5a*, *Zfp367*, and *Csrnp1*) with low expression but robust up-regulation upon PNI in rats and mice. Besides, these three TFs also showed up-regulated in the spinal cord after CNS injury, specifically *Arid5a* and *Csrnp1* that significantly up-regulated at as early as 3 h, suggesting their roles acting as a stress response regulator. Further temporal analysis showed the similar expression patterns of these three TFs with other pro-regenerative TFs (*e.g. Atf3*, *Sox11*, *Jun*, *Myc*) that robustly up-regulated at 1d when previously defined as the pre-regeneration stage^30^. These evidences highlighted their importance in axon regeneration. Except for the discussion of robust DEGs, we also screened candidate genes presenting species-preference manners (*e.g. Hamp*, *Serpina3n*, *Csrp3*, *Reg3b*, *Vip*, *Crisp3*, *Stac2*, *LOC688549* in rats; *Tmem88b*, *Gpr151*, *Anxa10*, *Cbr2*, *Plppr4*, *Lipn*, *Fgf3*, *Nts*, *Slc6a4* in mice). Analysis of these species-preference genes showed that distinct inflammation and immune response mechanism may exist between mouse and rat in response to injury and required further functional exploration. Specifically, we found lipid-related genes present in mouse-preference genes, including lipase (*Lipn*, *Liph*) and lipid phosphate phosphatase (*Plppr4*). A difference in lipid-related genes in Schwann cells from mouse and human after traumatic injury was also reported^32^. Reducing damaged elevated triacylglycerol and lipid phosphates promote axon regeneration^32,60^. Robust up-regulation of lipase and *Plppr4* may benefit axon regeneration in mice upon injury. Besides, we also explored the expression patterns of DEGs by cross-validation and highlighted two important timepoints for regeneration initiation (1d) and regenerating stages (3d) based on the available time-series expression profile. Finally but not the least, we identified four male-specific makers which could be used to infer the sex of public RNA-seq of rodent DRGs.

In summary, this study provided an important and integrated genome-wide landscape for gene expression change in rodent DRGs upon PNI, and cross-validation highlighted some less-studied TFs or genes involved in axon injury and regeneration for further investigation. More importantly, it provided new insights into evolutionarily conserved and specific molecular expression signatures upon injury which will greatly contribute to the neuroscience community. Besides, integrated resources of DRG expression upon PNI across species may provide new targets for translational approaches to treat nerve injury and NP.

## Methods

### Data collection

We searched keywords “dorsal root ganglion” and “injury” on the GEO or Sequence Read Archive (SRA) database for collecting public expression profiles of high throughput sequencing (RNA-seq) (last accessed on Dec 2021). Only datasets with public literature and mouse or rat models were discussed in this study. Sample information in each study was carefully and manually checked by confirming in the original paper and details were shown in (Supplementary Tables 1 and 2).

### Identification of orthologous relationship of protein-coding genes between mouse and rat

Protein sequences were firstly retrieved from NCBI (rat: mRatBN7.2) and Gencode (mouse: vM27) databases. The longest protein sequence for those genes with multiple isoforms was selected as the representative sequence for each gene. OrthoFinder pipeline^73^ was employed to detect orthologous relationships between mouse and rat with default parameters except for BLAST as an aligner for comparative analysis. Those genes with single-copy orthologous relationship or the same gene nomenclature were selected for comparison between mouse and rat in response to nerve injury.

### RNA-seq analysis

We performed the re-analysis of the public RNA-seq datasets using the uniform reference annotation and pipeline as we described in Xu *et al*.^74^. Simply as follows: FastQC software was used to check quality including adapter sequences and base quality distribution and then trimmed with Trimmomatic^75^ (v0.38). HISAT2^76^ (v2.1.0) was used to align clean reads against the reference genome with default parameters and the experiment type (stranded or not) was determined by RSeQC package^77^ (v4.0.0). To quantify gene expression, we employed the featureCount program implemented in SubRead package^78^ (v1.6.2) to call read count and then was normalized into RPKM (Reads Per Kilobase of transcript per Million reads mapped) or FPKM (Fragments Per Kilobase of transcript per Million reads mapped) in a single-end or paired-end sequencing mode. For differentially expressed genes (DEGs) compared to the control group (sham-operated or naïve), we employed DESeq. 2, and genes with |log_2_^fold-change^| ≥ log_2_^1.5^ and adjusted *P*-value ≤ 0.05 were defined as DEGs of datasets with biological replicates. For datasets without replicates, we employed CORNAS^79^ and genes with |log_2_^fold-change^| ≥ log_2_^1.5^. Only injury time points with significant up-regulation of *Atf3* were kept in the analysis of this study. Gene ranking was performed by calculating the π-value (-log_10_^(adjusted *P*-value)^ × log_2_^(Fold-change)^) in those datasets with biological replicates (if adjusted *P*-value < 1e^−20^ then adjusted *P*-value was set to 1e-20). For those datasets without biological replicates, gene ranking was ordered by log_2_^(Fold-change)^. The top 15 ranking genes in each comparison were extracted and then calculated their frequency of occurrence in models (spared nerve injury, sciatic nerve injury, and spinal nerve injury) from mice and rats separately. Then ggwordcloud R package was used for visualization of the frequency of top 15 ranking genes in four groups. The visualization of DEGs in each compared group was shown as a manhattan plot using the ggplot2 package. Heatmap visualization of LFC or expression values were conducted using the ComplexHeatmap package^80^.

### Identification of robust DEGs upon injury

To screen robust injury-related genes, we selected genes present in most of the compared groups in each injury model of rats or mice (*e.g*. 50%). For each DEG, we assigned distinct weights for up- (+1) and down-regulation (−1) in each comparison and summed them into a single score within a group (spared nerve injury or sciatic nerve injury from mice; sciatic nerve injury or spinal nerve injury from rats) as follows:

$${x}_{ij}=\left\{\begin{array}{l}1\;(up)\\ -1\left(down\right)\end{array}\right.;\;scor{e}_{i}={\sum }_{j=1}^{N}{x}_{ij}$$

where *x*_*ij*_ is the weight of a DEG (i) in the comparison (j) in a group and *score*_*i*_ is the summed weights of the DEG (i) in all comparisons (N) in a group.

To screen robust up- and down-regulated genes, DEGs with a |*score*|≥50% × *N* were defined as robust.

### Expression pattern analysis

Five studies investigated time-series profiles of DRG upon injury during the first week with at least three injury time points. To systematically understand expression patterns of DEGs and increase reliability, we employed the Mfuzz package^81^ to cluster DEGs into eight groups for each time-series dataset. Visualization of gene relationship in each cluster between SRP134051 and other datasets was performed using the NGenomeSyn (https://github.com/hewm2008/NGenomeSyn). To explore the potential functions of genes in each cluster, clusterProfiler^82^ was used to perform functional enrichment analysis.

### Web-based resource construction

To provide an easy exploration of gene expression change in diverse DRG profiles in this study, we used the shiny package and R program to build a website for convenient access (http://121.41.67.1:3838/DRGProfile/ and Figshare^83^).

## Supplementary information

Supplementary Figures

Supplementary Table1

Supplementary Table2

Supplementary Table3

Supplementary Table4

Supplementary Table5

Supplementary Table6

Supplementary Table7

Supplementary Table8

Supplementary Table9

## Data Availability

All sequencing data of DRG tissue/neurons following peripheral nerve injury discussed in this study could be freely retrieved from the public SRA database under accessions: D1 (SRP002416)^19,84^, D2 (SRP044030)^17,85^, D3 (SRP034868)^29,86^, D4 (SRP125336)^23,87^, D5 (SRP109547)^26,88^, D6 (SRP268785)^11,89^, D7 (SRP056393)^27,90^, D8 (SRP044619)^13,91^, D9 (SRP134051)^20,92^, D10 (SRP182089)^18,93^, D11 (SRP200823)^28,94^, D12 (SRP055201)^4,95^, D13 (SRP154895)^10,96^, D14 (SRP102543)^12,97^, D15 (SRP332955)^15,98^, D16 (SRP157873)^22,99^, D17 (SRP253717)^21,100^, D18 (SRP061708)^14,101^, D19 (SRP115543)^25,102^, D20 (SRP133622)^24,103^, D21 (SRP125336)^23,87^. For additional datasets supporting our conclusions: spinal cord injury (OEP000369)^35,104^, spinal cord development (SRP168574)^34,105^, sciatic nerve (SRP113121)^33,106^, single-cell sequencing of DRG neurons (SRP061708)^14,101^ and culture adult DRG neurons within 24 h after plating and embryonic DRG neurons (SRP055201)^4,95^. Uniformly processed gene expression profiles and visualization of genes of this study were easily accessed at our web-based DRGProfile (http://121.41.67.1:3838/DRGProfile/) as well as at the Figshare database^83^. To support data sharing and reusability, all fully processed individual datasets are available in the Figshare^83^. In addition, Supplementary Tables 2–9 are also available at the Figshare^83^ associated with this article.

## Code availability

The bioinformatics pipeline (reads trimming, mapping, quantification and differential expression) with a detailed list of command lines, and data and source code of DRGProfile shinyApp, were deposited on the repository Figshare^83^.

## References

[1] He Z, Jin Y (2016). Intrinsic Control of Axon Regeneration. Neuron.

[2] Colloca L (2017). Neuropathic pain. Nat Rev Dis Primers.

[3] Nascimento AI, Mar FM, Sousa MM (2018). The intriguing nature of dorsal root ganglion neurons: Linking structure with polarity and function. Prog Neurobiol.

[4] Tedeschi A (2016). The Calcium Channel Subunit Alpha2delta2 Suppresses Axon Regeneration in the Adult CNS. Neuron.

[5] Bauder, A. R. & Ferguson, T. A. Reproducible mouse sciatic nerve crush and subsequent assessment of regeneration by whole mount muscle analysis. *J Vis Exp*, e3606, 10.3791/3606 (2012). 10.3791/3606 PMC3376939 22395197

[6] Dowdall T, Robinson I, Meert TF (2005). Comparison of five different rat models of peripheral nerve injury. Pharmacol Biochem Behav.

[7] Liu S, Trapnell C (2016). Single-cell transcriptome sequencing: recent advances and remaining challenges. F1000Res.

[8] Ozsolak F, Milos PM (2011). RNA sequencing: advances, challenges and opportunities. Nat Rev Genet.

[9] Renthal W (2020). Transcriptional Reprogramming of Distinct Peripheral Sensory Neuron Subtypes after Axonal Injury. Neuron.

[10] Sun W (2020). A Transcriptomic Analysis of Neuropathic Pain in Rat Dorsal Root Ganglia Following Peripheral Nerve Injury. Neuromolecular Med.

[11] Kalinski AL (2020). Analysis of the immune response to sciatic nerve injury identifies efferocytosis as a key mechanism of nerve debridement. Elife.

[12] Hervera A (2018). Reactive oxygen species regulate axonal regeneration through the release of exosomal NADPH oxidase 2 complexes into injured axons. Nat Cell Biol.

[13] Motti D (2017). Identification of miRNAs involved in DRG neurite outgrowth and their putative targets. FEBS Lett.

[14] Hu G (2016). Single-cell RNA-seq reveals distinct injury responses in different types of DRG sensory neurons. Sci Rep.

[15] Chernov AV, Shubayev VI (2021). Sexual Dimorphism of Early Transcriptional Reprogramming in Dorsal Root Ganglia After Peripheral Nerve Injury. Front Mol Neurosci.

[16] Ahlstrom FHG (2021). Spared Nerve Injury Causes Sexually Dimorphic Mechanical Allodynia and Differential Gene Expression in Spinal Cords and Dorsal Root Ganglia in Rats. Mol Neurobiol.

[17] Laumet G (2015). G9a is essential for epigenetic silencing of K(+) channel genes in acute-to-chronic pain transition. Nat Neurosci.

[18] Carlin, D., Halevi, A. E., Ewan, E. E., Moore, A. M. & Cavalli, V. Nociceptor Deletion of Tsc2 Enhances Axon Regeneration by Inducing a Conditioning Injury Response in Dorsal Root Ganglia. *eNeuro* 6, ENEURO.0168-19.2019, 10.1523/ENEURO.0168-19.2019 (2019). 10.1523/ENEURO.0168-19.2019 PMC6595439 31182472

[19] Hammer P (2010). mRNA-seq with agnostic splice site discovery for nervous system transcriptomics tested in chronic pain. Genome Res.

[20] Perry RB, Hezroni H, Goldrich MJ, Ulitsky I (2018). Regulation of Neuroregeneration by Long Noncoding RNAs. Mol Cell.

[21] Wang XW (2020). Knocking Out Non-muscle Myosin II in Retinal Ganglion Cells Promotes Long-Distance Optic Nerve Regeneration. Cell Rep.

[22] Shin JE, Ha H, Kim YK, Cho Y, DiAntonio A (2019). DLK regulates a distinctive transcriptional regeneration program after peripheral nerve injury. Neurobiol Dis.

[23] Baskozos G (2019). Comprehensive analysis of long noncoding RNA expression in dorsal root ganglion reveals cell-type specificity and dysregulation after nerve injury. Pain.

[24] Parisien M (2019). Genetic pathway analysis reveals a major role for extracellular matrix organization in inflammatory and neuropathic pain. Pain.

[25] Cobos EJ (2018). Mechanistic Differences in Neuropathic Pain Modalities Revealed by Correlating Behavior with Global Expression Profiling. Cell Rep.

[26] Stephens KE (2019). Sex differences in gene regulation in the dorsal root ganglion after nerve injury. BMC Genomics.

[27] Omura T (2015). Robust Axonal Regeneration Occurs in the Injured CAST/Ei Mouse CNS. Neuron.

[28] Mao S (2019). Circ-Spidr enhances axon regeneration after peripheral nerve injury. Cell Death Dis.

[29] Perkins JR (2014). A comparison of RNA-seq and exon arrays for whole genome transcription profiling of the L5 spinal nerve transection model of neuropathic pain in the rat. Mol Pain.

[30] Li S (2015). The transcriptional landscape of dorsal root ganglia after sciatic nerve transection. Sci Rep.

[31] Chandran V (2016). A Systems-Level Analysis of the Peripheral Nerve Intrinsic Axonal Growth Program. Neuron.

[32] Meyer Zu Reckendorf S (2020). Lipid metabolism adaptations are reduced in human compared to murine Schwann cells following injury. Nat Commun.

[33] Yi S (2015). Deep Sequencing and Bioinformatic Analysis of Lesioned Sciatic Nerves after Crush Injury. PLoS One.

[34] Yang J (2021). Developmental Temporal Patterns and Molecular Network Features in the Transcriptome of Rat Spinal Cord. Engineering.

[35] Yu B (2019). The Landscape of Gene Expression and Molecular Regulation Following Spinal Cord Hemisection in Rats. Front Mol Neurosci.

[36] Tsujino H (2000). Activating transcription factor 3 (ATF3) induction by axotomy in sensory and motoneurons: A novel neuronal marker of nerve injury. Mol Cell Neurosci.

[37] Xiao Y (2014). A novel significance score for gene selection and ranking. Bioinformatics.

[38] Murata Y (2015). Protein tyrosine phosphatase SAP-1 protects against colitis through regulation of CEACAM20 in the intestinal epithelium. Proc Natl Acad Sci USA.

[39] Belardin L (2019). Cysteine-rich secretory protein 3: inflammation role in adult varicocoele. Andrology.

[40] Xia LP (2021). GPR151 in nociceptors modulates neuropathic pain via regulating P2X3 function and microglial activation. Brain.

[41] Li F, Xue ZY, Liu X, Bai G, Wang YL (2018). Annexin A10 contributes to chronic constrictive injury-induced pain through activating ERK1/2 signalling in rats. Int J Neurosci.

[42] Nyati KK, Kishimoto T (2021). Recent Advances in the Role of Arid5a in Immune Diseases and Cancer. Front Immunol.

[43] Macdonald CD (2018). Cytokine-induced cysteine- serine-rich nuclear protein-1 (CSRNP1) selectively contributes to MMP1 expression in human chondrocytes. PLoS One.

[44] Feijoo CG, Sarrazin AF, Allende ML, Glavic A (2009). Cystein-serine-rich nuclear protein 1, Axud1/Csrnp1, is essential for cephalic neural progenitor proliferation and survival in zebrafish. Dev Dyn.

[45] Simoes-Costa M, Stone M, Bronner ME (2015). Axud1 Integrates Wnt Signaling and Transcriptional Inputs to Drive Neural Crest Formation. Dev Cell.

[46] Weninger SC (1999). Stress-induced behaviors require the corticotropin-releasing hormone (CRH) receptor, but not CRH. Proc Natl Acad Sci USA.

[47] D’Este G (2022). Latrotoxin-Induced Neuromuscular Junction Degeneration Reveals Urocortin 2 as a Critical Contributor to Motor Axon Terminal Regeneration. Int J Mol Sci.

[48] Byrne AB (2016). Inhibiting poly(ADP-ribosylation) improves axon regeneration. Elife.

[49] Latremoliere A (2015). Reduction of Neuropathic and Inflammatory Pain through Inhibition of the Tetrahydrobiopterin Pathway. Neuron.

[50] Kalinski AL (2015). mRNAs and Protein Synthetic Machinery Localize into Regenerating Spinal Cord Axons When They Are Provided a Substrate That Supports Growth. J Neurosci.

[51] Vicuna L (2015). The serine protease inhibitor SerpinA3N attenuates neuropathic pain by inhibiting T cell-derived leukocyte elastase. Nat Med.

[52] Berta T (2017). Gene Expression Profiling of Cutaneous Injured and Non-Injured Nociceptors in SNI Animal Model of Neuropathic Pain. Sci Rep.

[53] Stam FJ (2007). Identification of candidate transcriptional modulators involved in successful regeneration after nerve injury. Eur J Neurosci.

[54] Li J (2020). Nerve Injury-Induced Neuronal PAP-I Maintains Neuropathic Pain by Activating Spinal Microglia. J Neurosci.

[55] Lu CC, Lu YY, Tsai HP, Wu CH (2022). The Contribution of TSLP Activation to Hyperalgesia in Dorsal Root Ganglia Neurons of a Rat. Int J Mol Sci.

[56] Gemes G (2012). Painful nerve injury increases plasma membrane Ca2+-ATPase activity in axotomized sensory neurons. Mol Pain.

[57] Kingston R, Amin D, Misra S, Gross JM, Kuwajima T (2021). Serotonin transporter-mediated molecular axis regulates regional retinal ganglion cell vulnerability and axon regeneration after nerve injury. PLoS Genet.

[58] Liu K (2020). FGF3 from the Hypothalamus Regulates the Guidance of Thalamocortical Axons. Dev Neurosci.

[59] Jungnickel J, Gransalke K, Timmer M, Grothe C (2004). Fibroblast growth factor receptor 3 signaling regulates injury-related effects in the peripheral nervous system. Mol Cell Neurosci.

[60] Brauer AU (2003). A new phospholipid phosphatase, PRG-1, is involved in axon growth and regenerative sprouting. Nat Neurosci.

[61] Kage H (2014). Claudin 4 knockout mice: normal physiological phenotype with increased susceptibility to lung injury. Am J Physiol Lung Cell Mol Physiol.

[62] Gu P (2021). Genetically blocking HPD via CRISPR-Cas9 protects against lethal liver injury in a pig model of tyrosinemia type I. Mol Ther Methods Clin Dev.

[63] Zhou YF (2017). Hepcidin Protects Neuron from Hemin-Mediated Injury by Reducing Iron. Front Physiol.

[64] Levin E (2019). Muscle LIM Protein Is Expressed in the Injured Adult CNS and Promotes Axon Regeneration. Cell Rep.

[65] Yang C (2020). Rewiring Neuronal Glycerolipid Metabolism Determines the Extent of Axon Regeneration. Neuron.

[66] Israeli S (2011). A mutation in LIPN, encoding epidermal lipase N, causes a late-onset form of autosomal-recessive congenital ichthyosis. Am J Hum Genet.

[67] Moore DL (2009). KLF family members regulate intrinsic axon regeneration ability. Science.

[68] Aillaud C (2017). Vasohibins/SVBP are tubulin carboxypeptidases (TCPs) that regulate neuron differentiation. Science.

[69] Wu J (2017). Neuroprotective effects of sulfiredoxin-1 during cerebral ischemia/reperfusion oxidative stress injury in rats. Brain Res Bull.

[70] Gu X (2021). Biodegradable Materials and the Tissue Engineering of Nerves. Engineering.

[71] Nguyen MQ, von Buchholtz LJ, Reker AN, Ryba NJ, Davidson S (2021). Single-nucleus transcriptomic analysis of human dorsal root ganglion neurons. Elife.

[72] Ray P (2018). Comparative transcriptome profiling of the human and mouse dorsal root ganglia: an RNA-seq-based resource for pain and sensory neuroscience research. Pain.

[73] Emms DM, Kelly S (2019). OrthoFinder: phylogenetic orthology inference for comparative genomics. Genome Biol.

[74] Xu L (2020). Speciation and adaptive evolution reshape antioxidant enzymatic system diversity across the phylum Nematoda. BMC Biol.

[75] Bolger AM, Lohse M, Usadel B (2014). Trimmomatic: a flexible trimmer for Illumina sequence data. Bioinformatics.

[76] Kim D, Paggi JM, Park C, Bennett C, Salzberg SL (2019). Graph-based genome alignment and genotyping with HISAT2 and HISAT-genotype. Nat Biotechnol.

[77] Wang L, Wang S, Li W (2012). RSeQC: quality control of RNA-seq experiments. Bioinformatics.

[78] Liao Y, Smyth GK, Shi W (2013). The Subread aligner: fast, accurate and scalable read mapping by seed-and-vote. Nucleic Acids Res.

[79] Low JZB, Khang TF, Tammi MT (2017). CORNAS: coverage-dependent RNA-Seq analysis of gene expression data without biological replicates. BMC Bioinformatics.

[80] Gu Z, Eils R, Schlesner M (2016). Complex heatmaps reveal patterns and correlations in multidimensional genomic data. Bioinformatics.

[81] Kumar L, M EF (2007). Mfuzz: a software package for soft clustering of microarray data. Bioinformation.

[82] Yu G, Wang LG, Han Y, He QY (2012). clusterProfiler: an R package for comparing biological themes among gene clusters. OMICS.

[83] Yang J (2022). figshare.

[84] (2010). NCBI Sequence Read Archive.

[85] (2015). NCBI Sequence Read Archive.

[86] (2015). NCBI Sequence Read Archive.

[87] (2019). NCBI Sequence Read Archive.

[88] (2019). NCBI Sequence Read Archive.

[89] (2021). NCBI Sequence Read Archive.

[90] (2015). NCBI Sequence Read Archive.

[91] (2017). NCBI Sequence Read Archive.

[92] (2018). NCBI Sequence Read Archive.

[93] (2019). NCBI Sequence Read Archive.

[94] (2019). NCBI Sequence Read Archive.

[95] (2016). NCBI Sequence Read Archive.

[96] (2020). NCBI Sequence Read Archive.

[97] (2018). NCBI Sequence Read Archive.

[98] (2021). NCBI Sequence Read Archive.

[99] (2019). NCBI Sequence Read Archive.

[100] (2020). NCBI Sequence Read Archive.

[101] (2016). NCBI Sequence Read Archive.

[102] (2018). NCBI Sequence Read Archive.

[103] (2019). NCBI Sequence Read Archive.

[104] The landscape of gene expression and molecular regulation following spinal cord hemisection in rats. The National Omics Data Encyclopedia, https://www.biosino.org/node/project/detail/OEP000369 (2019). 10.3389/fnmol.2019.00287 PMC6883948 31824262

[105] (2019). NCBI Sequence Read Archive.

[106] (2017). NCBI Sequence Read Archive.

