# Supplementary material for: Integrated analyses reveal evolutionarily conserved and specific injury response genes in dorsal root ganglion
Source: Sci Data. 2022 Nov 2;9:666. doi: 10.1038/s41597-022-01783-8 (PMC9630366; doi:10.1038/s41597-022-01783-8)
Supplement: Supplementary file 1 — Supplementary Figures [file 41597_2022_1783_MOESM1_ESM.docx]

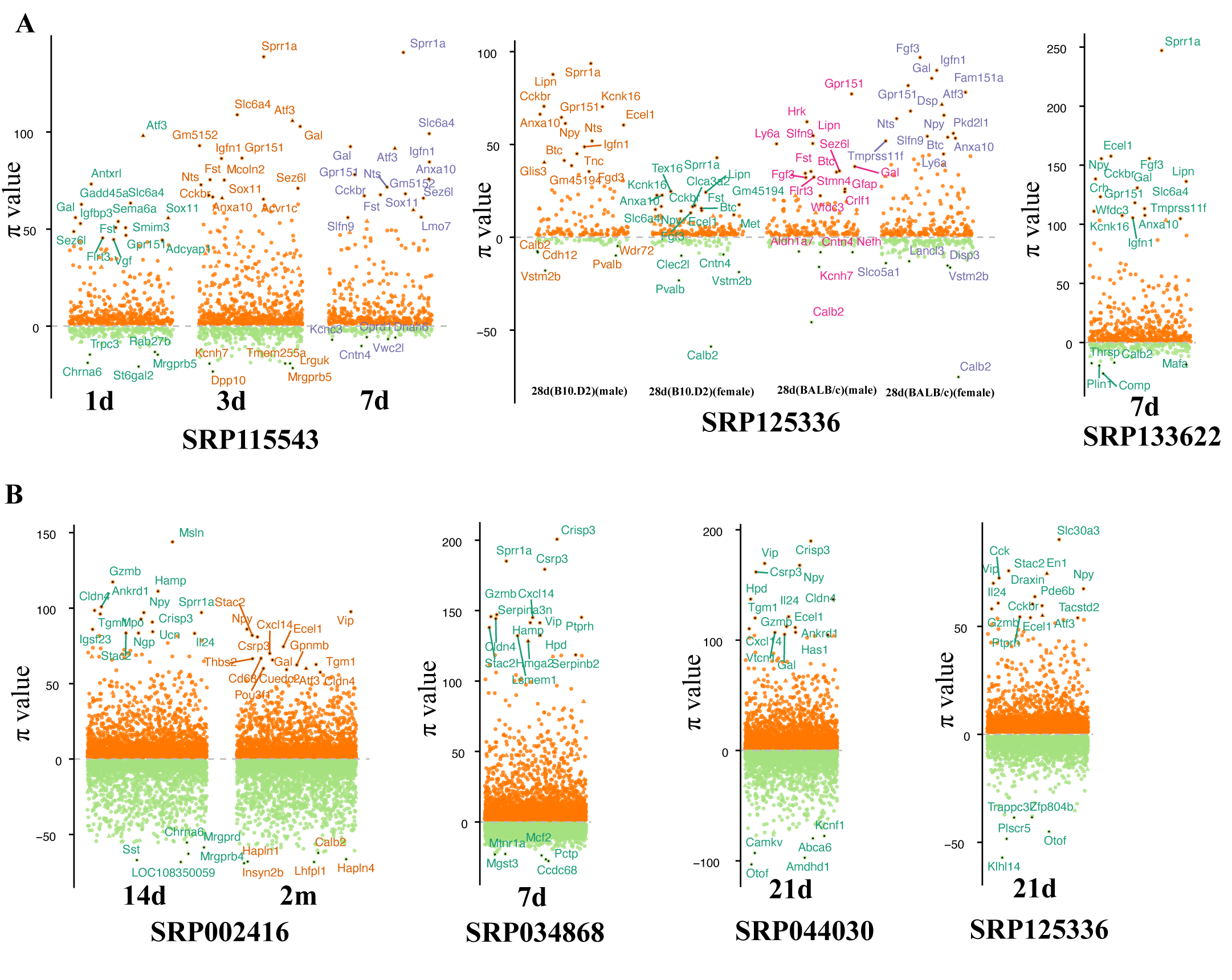


**Supplementary Figure 1. DEG ranking for each comparison in each dataset from SNI (A) and SpNI models (B)**. orange and green points indicated up-regulated and down-regulated DEGs respectively. The top 15 up-regulated and top 5 down-regulated DEGs were labeled and colors indicated different comparisons in a dataset.


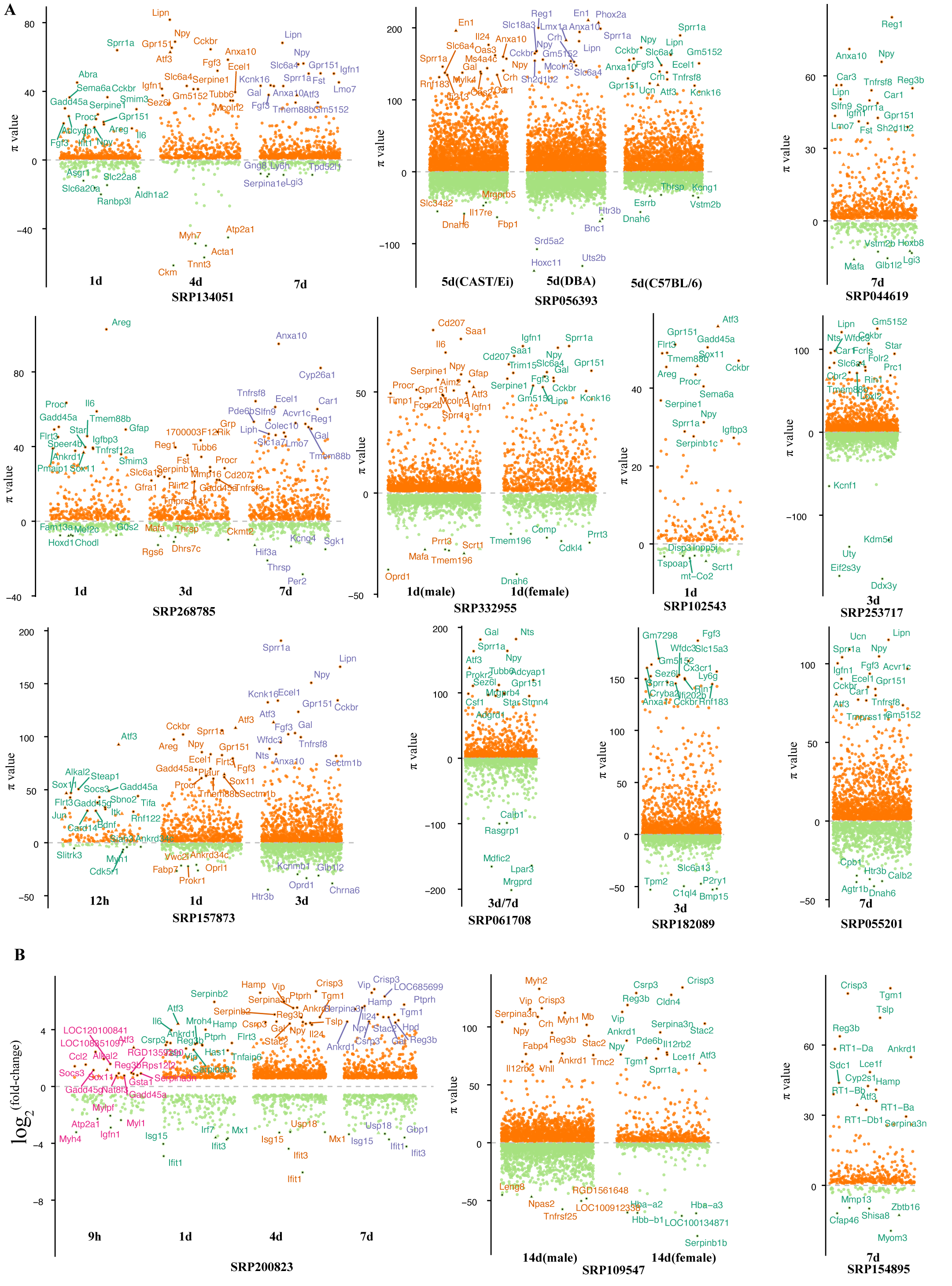


**Supplementary Figure 2. DEG ranking for each comparison in each ScNI dataset from mouse (A) and rat models (B)**. orange and green points indicated up-regulated and down-regulated DEGs respectively. The top 15 up-regulated and top 5 down-regulated DEGs were labeled and colors indicated different comparisons in a dataset.


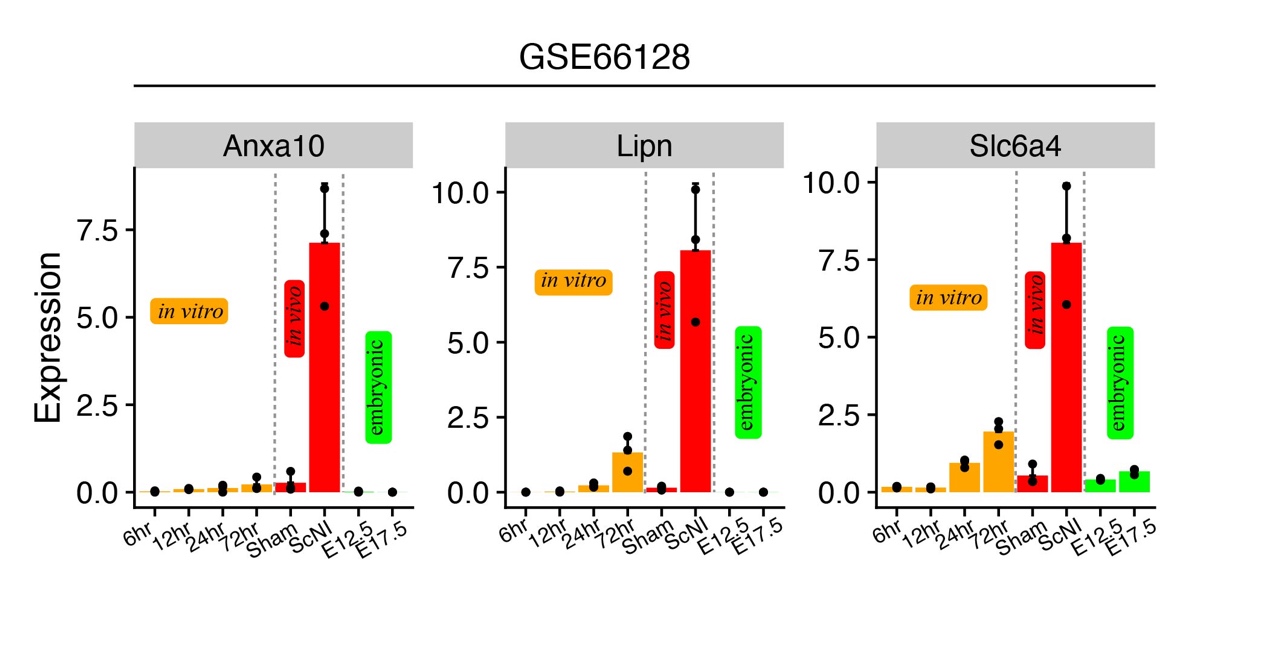


**Supplementary Figure 3. The expression of genes (*Anxa3*, *Lipn*, and *Slc6a4*) in DRG neurons collected from in *vitro* after plating, or sciatic nerve crush injury (ScNI) in *vivo*, or embryonic mice.**

**
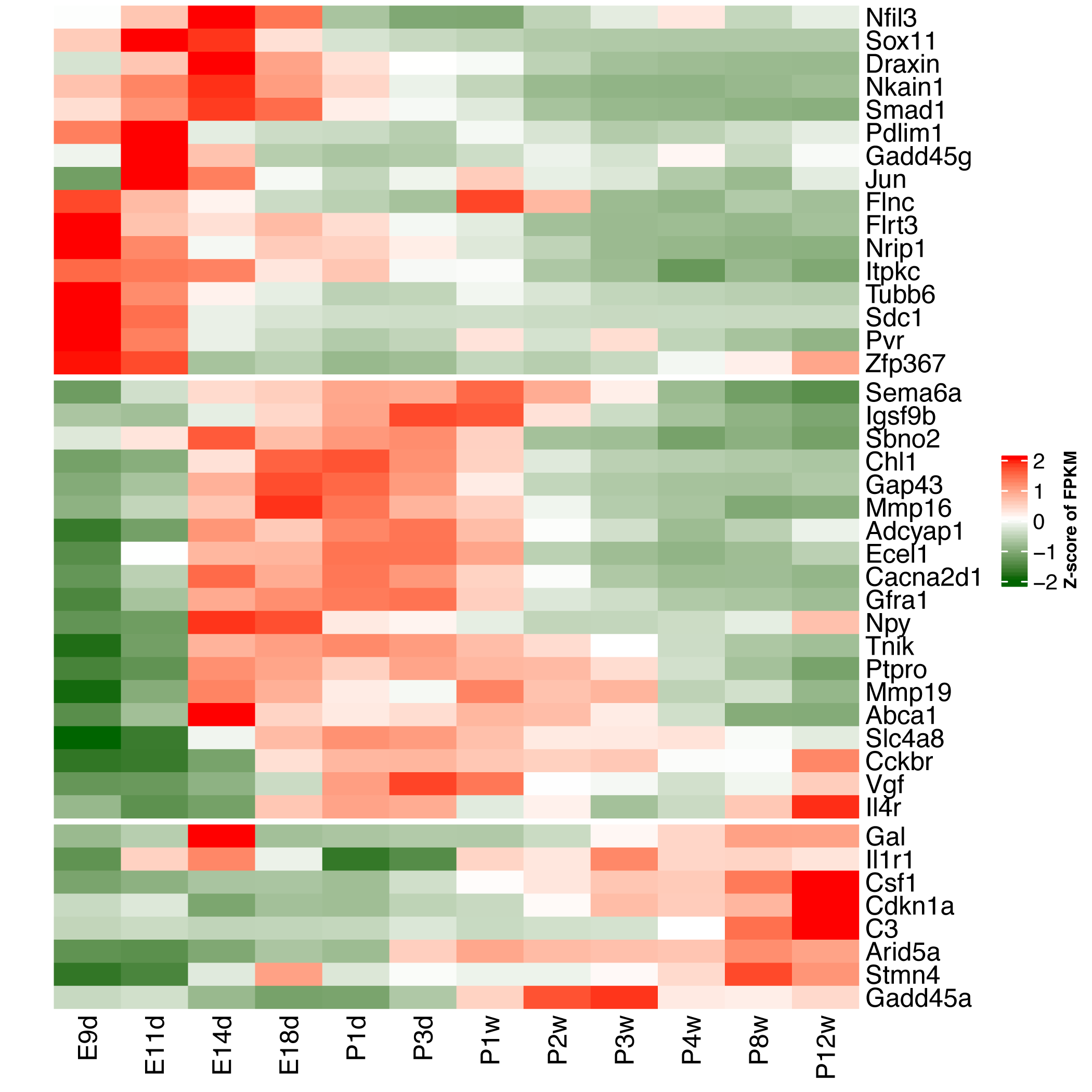
**

**Supplementary Figure 4. Expression profiles of 53 robust upregulated genes in spinal cord development datasets^1^.** Only genes with average expression ≥1 were shown.

**
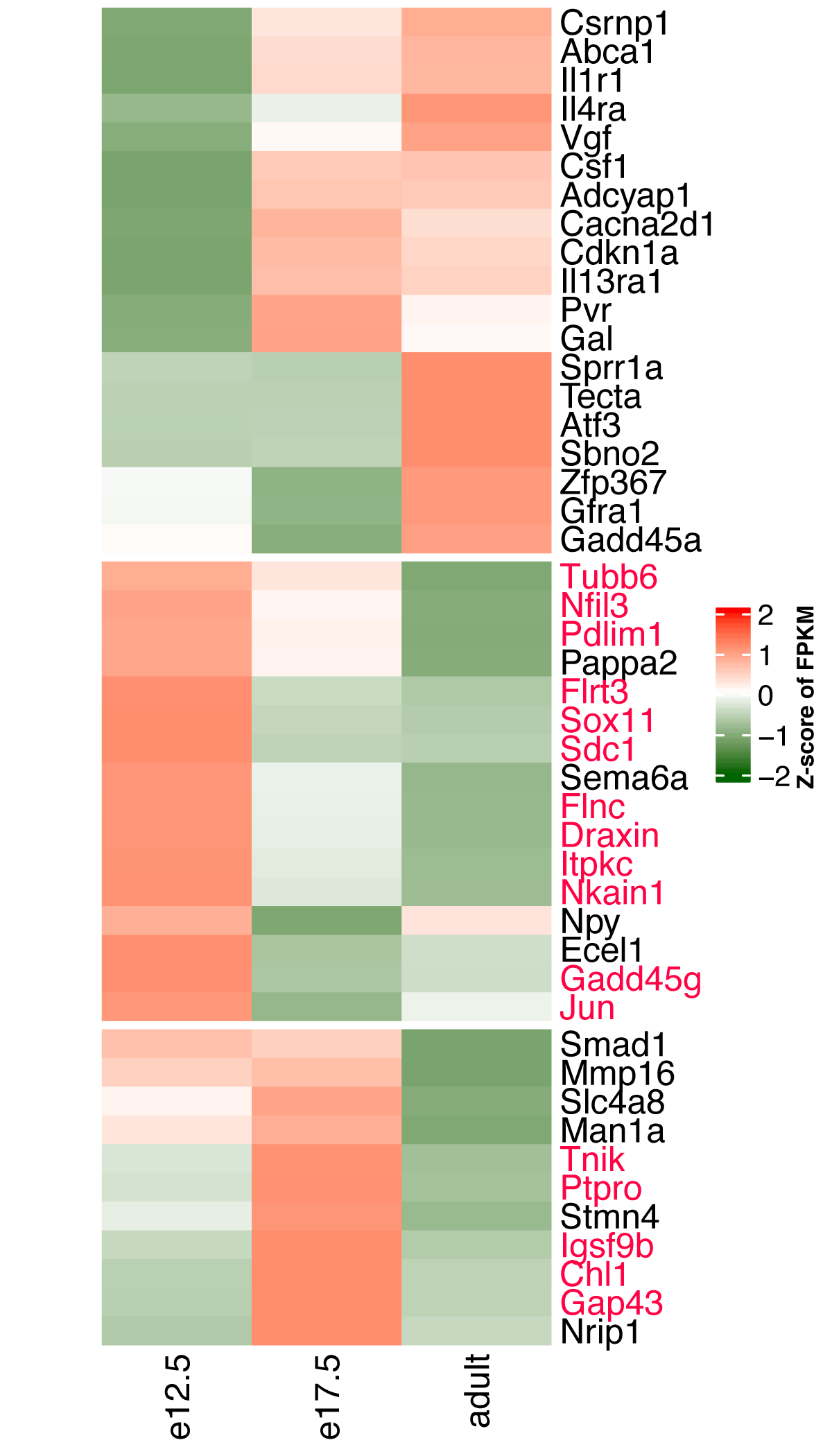
**

**Supplementary Figure 5. Expression profiles of 53 robust upregulated genes in DRG development datasets^2^.** Only genes with average expression ≥1 were shown. Red texts indicated genes highly expressed at the embryonic stage with similar expression patterns with developmental spinal cord profiles.

**
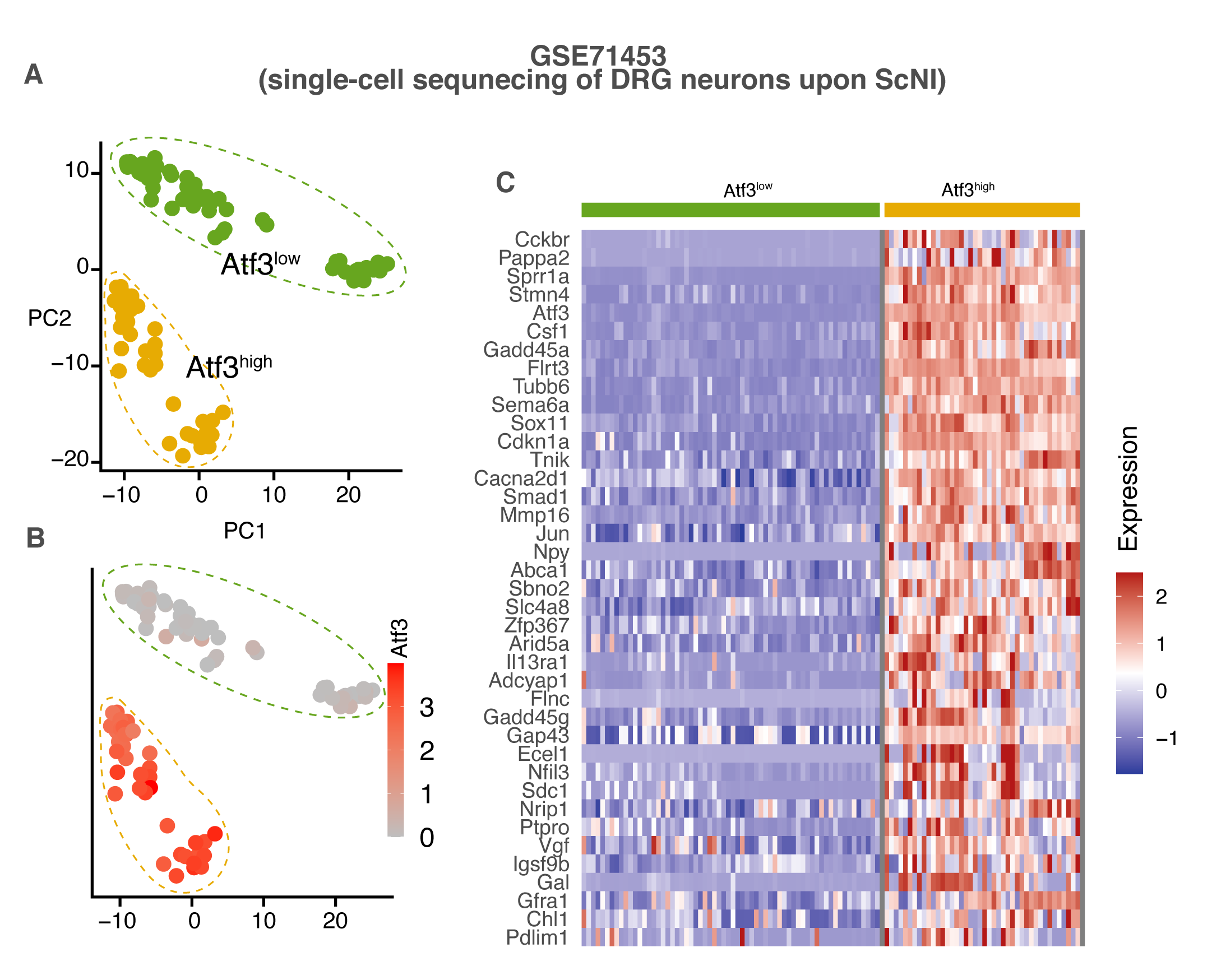
**

**Supplementary Figure 6. Differential expression of robust DEGs in injured DRG neurons. A,** PCA visualization of sample distribution from the GSE71453 dataset^3^. **B**, Expression of *Atf3* across samples. **C**, differential expression of robust DEG identified in **Fig. 2g** in the main text by comparing cells with high expression of *Atf3* to cells with low expression of *Atf3*.

**
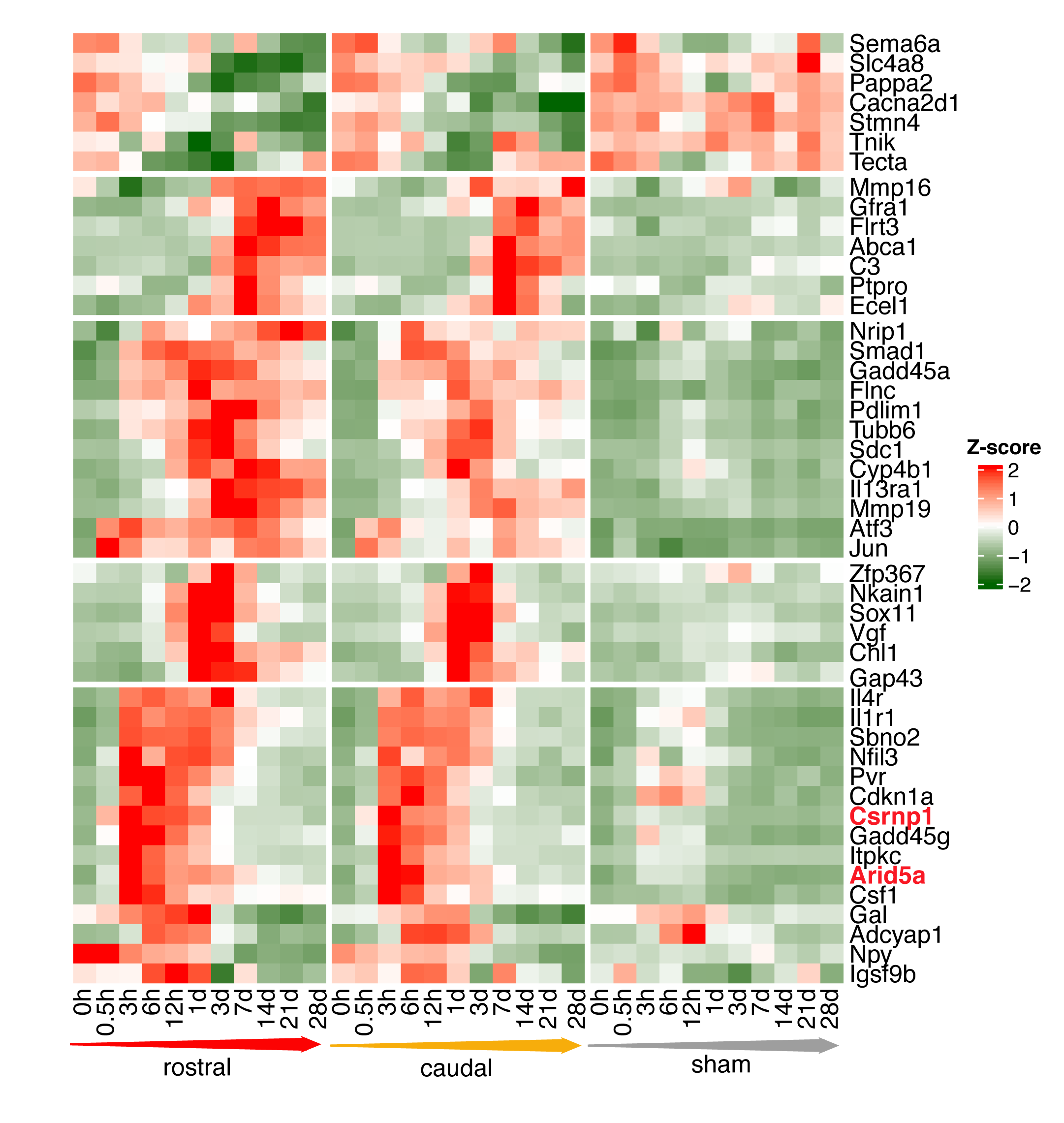
**

**Supplementary Figure 7. Expression profiles of 53 robust upregulated genes in spinal cord injury datasets^4^.** Only genes with average expression ≥1 were shown.

**
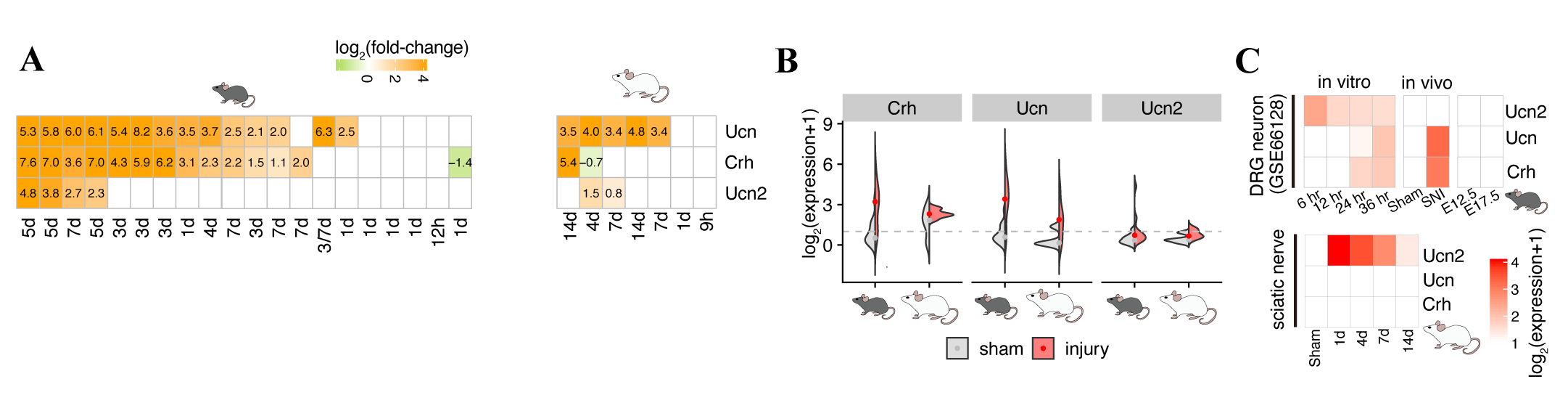
**

**Supplementary Figure 8. Expression of *Crh* family in rat and mouse upon PNI.** Dataset of DRG collected from in vitro and in vivo and embryonic stages were retrieved from the study^2^. Dataset of sciatic nerve upon crush injury was retrieved from the study^5^.

**
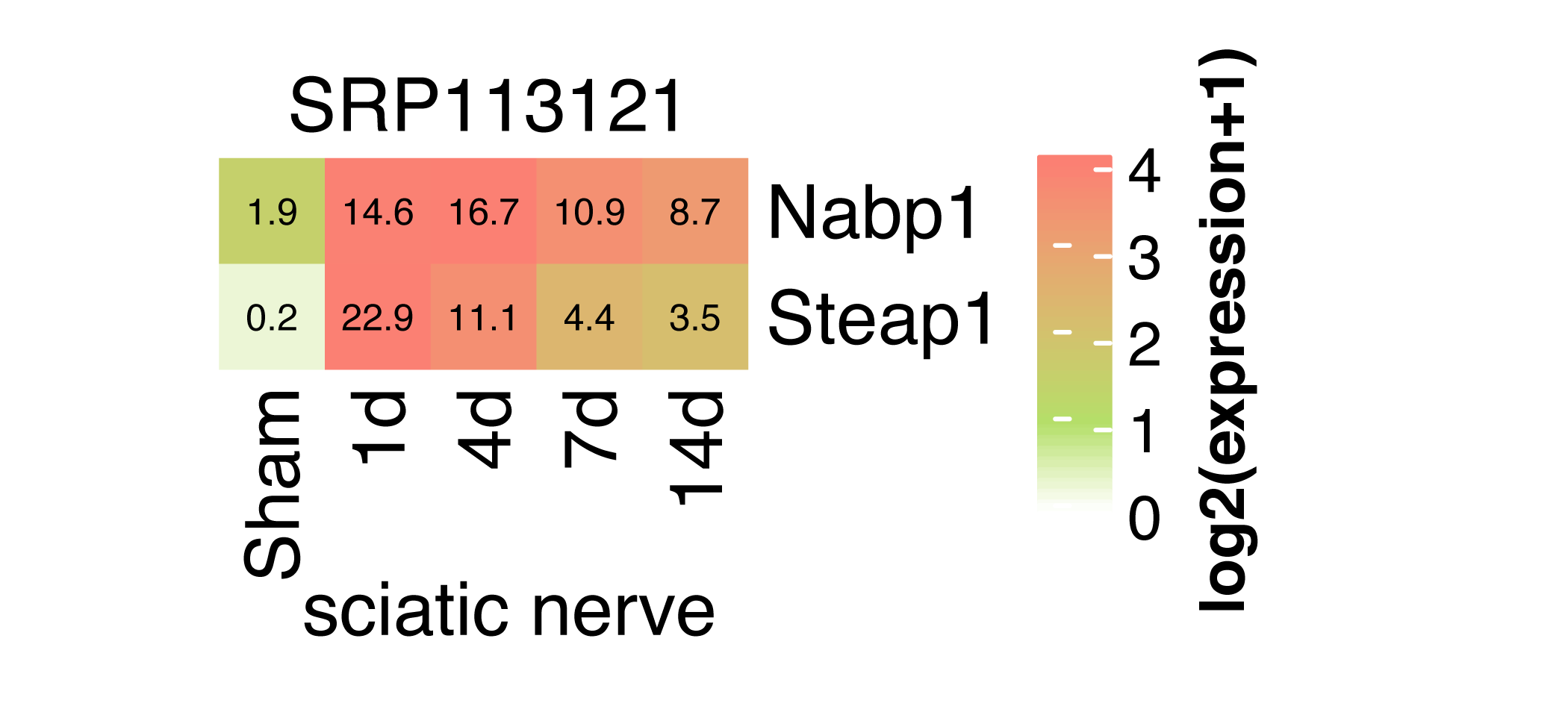
**

**Supplementary Figure 9. Expression of two genes identified in Fig. 3 in the main text in sciatic nerve upon crush injury.** data was retrieved from NCBI under accession SRP113121^5^.


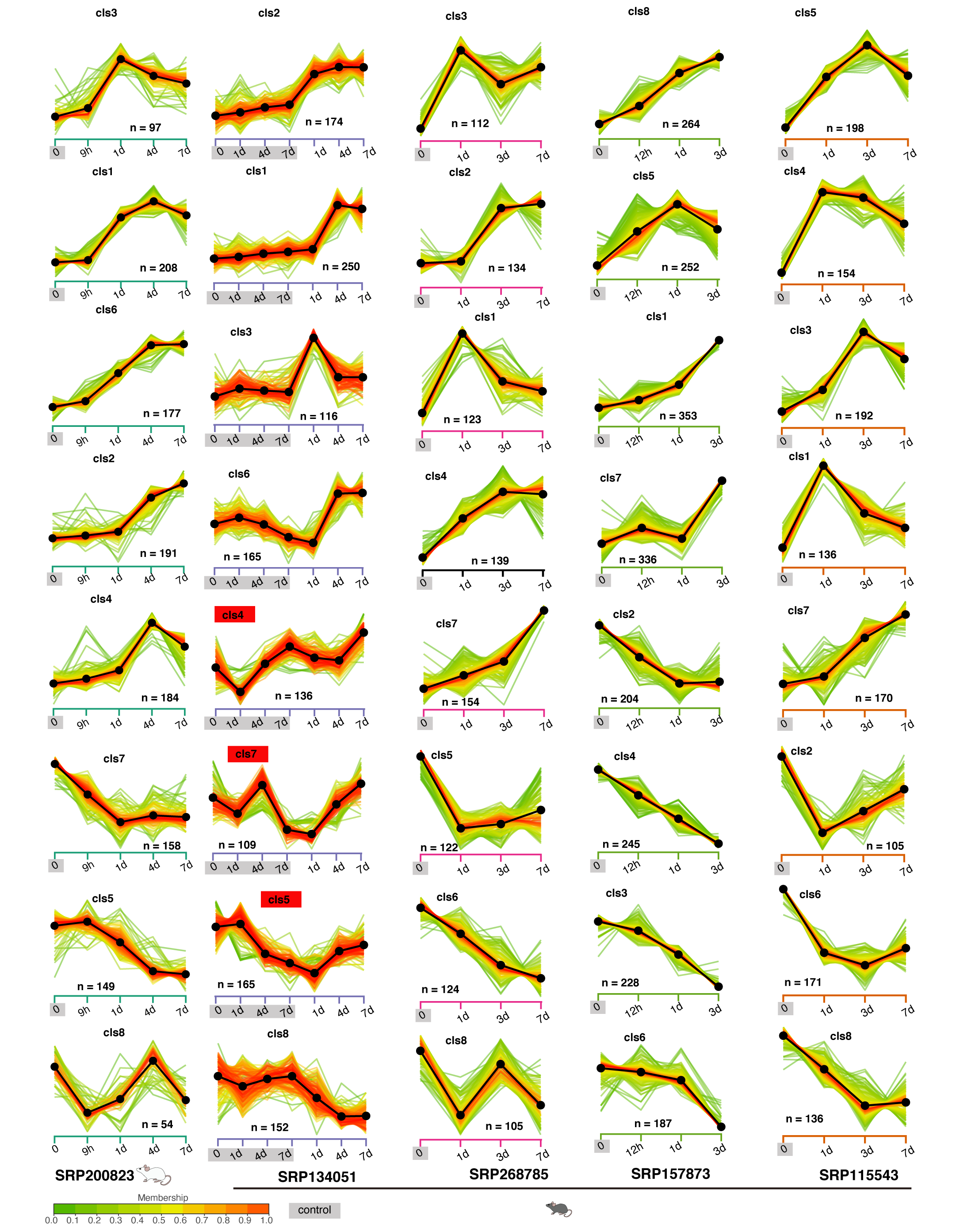


**Supplementary Figure 10. Expression patterns of each cluster among five time-series datasets**. Three clusters (red box) of the reference dataset (SRP134051) showed obvious expression change within the contralateral across time points post-injury which may be unrelated with nerve injury response.

**
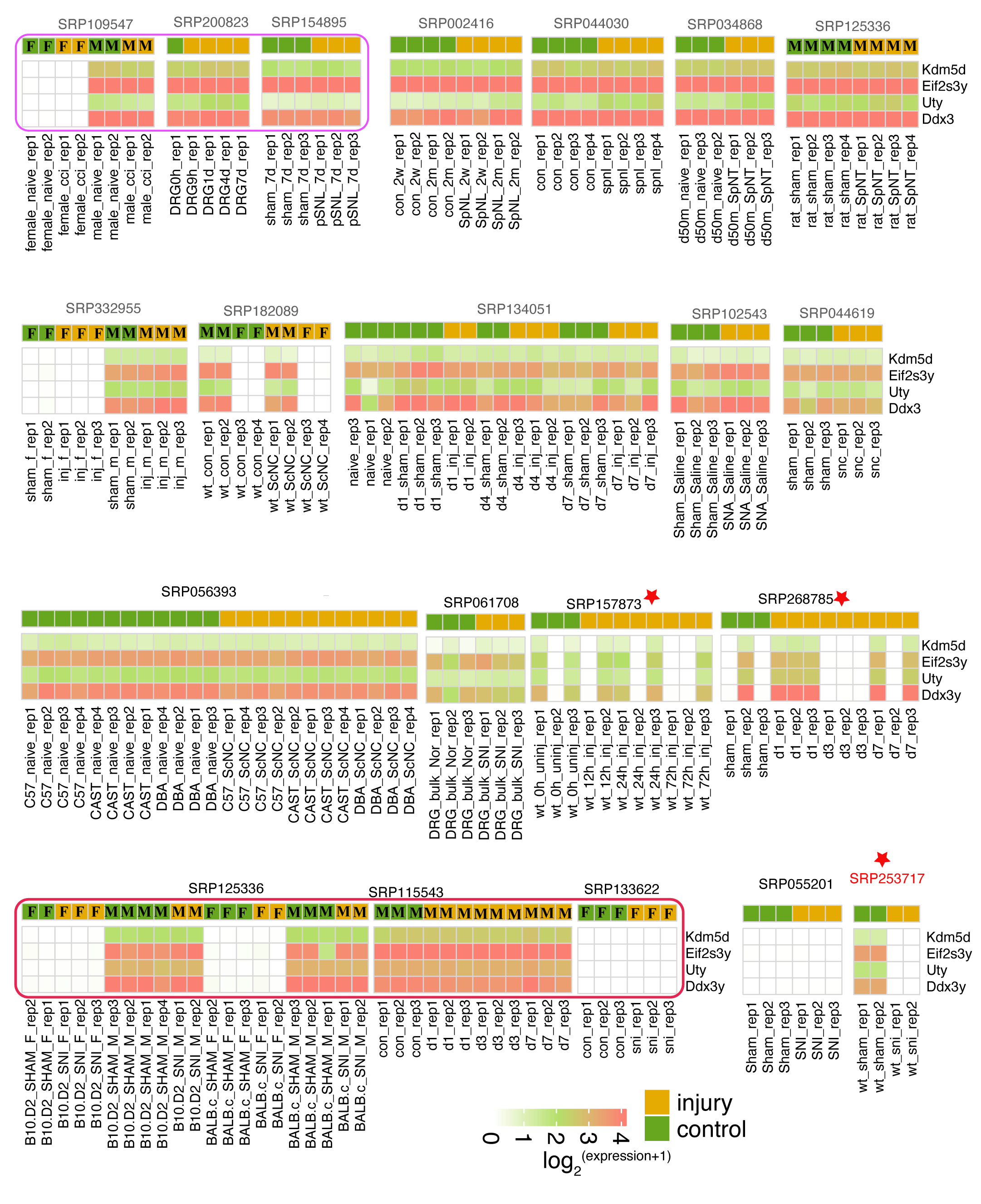
**

**Supplementary Figure 11. Expression of four male-specific genes in RNA-seq dataset discussed in this study.** Star indicated inconsistent sexual composition in a study.

1. Yang, J.*, et al.* Developmental Temporal Patterns and Molecular Network Features in the Transcriptome of Rat Spinal Cord. *Engineering* **7**, 1592-1602 (2021).

2. Tedeschi, A.*, et al.* The Calcium Channel Subunit Alpha2delta2 Suppresses Axon Regeneration in the Adult CNS. *Neuron* **92**, 419-434 (2016).

3. Hu, G.*, et al.* Single-cell RNA-seq reveals distinct injury responses in different types of DRG sensory neurons. *Sci Rep* **6**, 31851 (2016).

4. Yu, B.*, et al.* The Landscape of Gene Expression and Molecular Regulation Following Spinal Cord Hemisection in Rats. *Front Mol Neurosci* **12**, 287 (2019).

5. Yi, S.*, et al.* Deep Sequencing and Bioinformatic Analysis of Lesioned Sciatic Nerves after Crush Injury. *PLoS One* **10**, e0143491 (2015).
